# An empirical study on 209 networks of treatments revealed intransitivity to be common and multiple statistical tests suboptimal to assess transitivity

**DOI:** 10.1186/s12874-024-02436-7

**Published:** 2024-12-16

**Authors:** Loukia M. Spineli

**Affiliations:** Midwifery Research and Education Unit (OE 9210), Hannover Medical School, Carl-Neuberg-Straße 1, Hannover, 30625 Germany

**Keywords:** Systematic review, Network meta-analysis, Transitivity, Heterogeneity, Dissimilarity

## Abstract

**Background:**

Transitivity assumption is the cornerstone of network meta-analysis (NMA). Investigating the plausibility of transitivity can unveil the credibility of NMA results. The commonness of transitivity was examined based on study dissimilarities regarding several study-level aggregate clinical and methodological characteristics reported in the systematic reviews. The present study also demonstrated the disadvantages of using multiple statistical tests to assess transitivity and compared the conclusions drawn from multiple statistical tests with those from the approach of study dissimilarities for transitivity assessment.

**Methods:**

An empirical study was conducted using 209 published systematic reviews with NMA to create a database of study-level aggregate clinical and methodological characteristics found in the *tracenma* R package. For each systematic review, the network of the primary outcome was considered to create a dataset with extracted study-level aggregate clinical and methodological characteristics reported in the systematic review that may act as effect modifiers. Transitivity was evaluated by calculating study dissimilarities based on the extracted characteristics to provide a measure of overall dissimilarity within and between the observed treatment comparisons. Empirically driven thresholds of low dissimilarity were employed to determine the proportion of datasets with evidence of likely intransitivity. One-way ANOVA and chi-squared test were employed for each characteristic to investigate comparison dissimilarity at a significance level of 5%.

**Results:**

Study dissimilarities covered a wide range of possible values across the datasets. A ‘likely concerning’ extent of study dissimilarities, both intra-comparison and inter-comparison, dominated the analysed datasets. Using a higher dissimilarity threshold, a ‘likely concerning’ extent of study dissimilarities persisted for objective outcomes but decreased substantially for subjective outcomes. A likely intransitivity prevailed in all datasets; however, using a higher dissimilarity threshold resulted in few networks with transitivity for semi-objective and subjective outcomes. Statistical tests were feasible in 127 (61%) datasets, yielding conflicting conclusions with the approach of study dissimilarities in many datasets.

**Conclusions:**

Study dissimilarity, manifested from variations in the effect modifiers’ distribution across the studies, should be expected and properly quantified. Measuring the overall study dissimilarity between observed comparisons and comparing it with a proper threshold can aid in determining whether concerns of likely intransitivity are warranted.

**Supplementary Information:**

The online version contains supplementary material available at 10.1186/s12874-024-02436-7.

## Background

Since the introduction of the confidence profile method [1] and the adjusted indirect comparisons [2], the stocktaking of the research in the evidence synthesis field has marked an explosive rise in published systematic reviews with network meta-analysis (NMA), the extension of pairwise meta-analysis to multiple treatments [3–5]. The rapid evolution of the NMA methodology and the availability of software may have been the driving force behind the explosive publication record of systematic reviews with multiple treatments [6–9].

The various research areas of the NMA methodology have been granted with a different rate of advancement, with most of the emerging research focusing on model development of different complexity for treatment effects, consistency assessment between direct and indirect sources of evidence, bias adjustment methods and quality assessment of the NMA results [8–10]. Transitivity, the ability to validly learn about a comparison indirectly (i.e., estimate CB comparison via the BA and CA studies), has also received great attention for being the cornerstone of the NMA framework and the conceptual formulation of the consistency assumption [11, 12]. However, transitivity is primarily grounded on *conceptual* homogeneity across the evidence base, making the statistical evaluation of this assumption not straightforward [11]. Although the comparability of important effect modifiers across the (treatment) comparisons has been highlighted as pivotal to transitivity assessment [11–14], the relevant literature lacks transparency on how this comparability can be implemented, inviting limited attention to the transitivity assumption from systematic review authors [15].

Currently, authors of systematic reviews typically assess the transitivity assumption narratively by stating whether the investigated clinical and methodological characteristics are comparable across the studies or observed comparisons without providing further information [15]. Transparent evaluation of transitivity is sparse [15], and pertains to testing the association between each characteristic and the observed comparisons using analysis of variance (ANOVA), Kruskal-Wallis test or chi-squared test [16–25] and visualising the distribution of each effect modifier across the observed comparisons [14, 26]. Though they invite objectivity, statistical tests are sensitive to multiplicity issues and low power when the evidence is sparse or missing in many studies or comparisons. Visualisation is overly subjective and requires sufficient evidence to represent the data distribution.

It was recently proposed to use study dissimilarities to assess the transitivity assumption in a connected network of treatments [27]. This approach does not involve statistical testing but a simple measure to gauge the dissimilarity between observed comparisons based on study-level aggregate clinical and methodological characteristics that act as effect modifiers. Then, using an empirically-driven threshold of low dissimilarity, analysts can detect sets of comparisons that mismatch with most comparisons regarding their effect modifiers’ distribution, potentially compromising the transitivity in the network: pairs of comparisons associated with a dissimilarity that exceeds the threshold should be scrutinised to determine whether intransitivity concerns are justified.

The present empirical study primarily aims to uncover the extent of comparison dissimilarity and commonness of likely intransitivity in several connected networks by measuring study dissimilarity. Although statistical tests for transitivity assessment are not as common as the narrative evaluation of the assumption, they are a viable (and tempting) option when there is sufficient information to summarise the clinical and methodological characteristics for each comparison in a table. Therefore, the present study also aims to demonstrate the disadvantages of using multiple statistical tests to assess the transitivity assumption. Multiple statistical tests were also juxtaposed with the approach of study dissimilarities to compare the conclusions concerning likely intransitivity. To these intentions, the *tracenma* R package was created [28], which is the first database containing several study-level aggregate clinical and methodological characteristics (that may act as effect modifiers) extracted from published systematic reviews with NMA.

## Methods

### The database

The *nmadb* database [29] was used to construct the present database, which can be found in the *tracenma* R package [28]. The *nmadb* database contains 453 NMAs extracted from systematic reviews with at least four treatments published between 1999 and April 14, 2015 [4, 29, 30]. This database has available data for 286 (63%) networks. Of those, 72 (25%) were excluded for several reasons, yielding 214 eligible networks (from now on called *datasets*) for the present study. Additional file 1: Table S1 describes the network selection process to build the analysis database for the present article [29]. Additional file 2: Methods A includes information on the extracted characteristics of the present study and extraction challenges encountered [10, 29, 31–34].

### Using study dissimilarities to transitivity evaluation

**Fig. 1:**
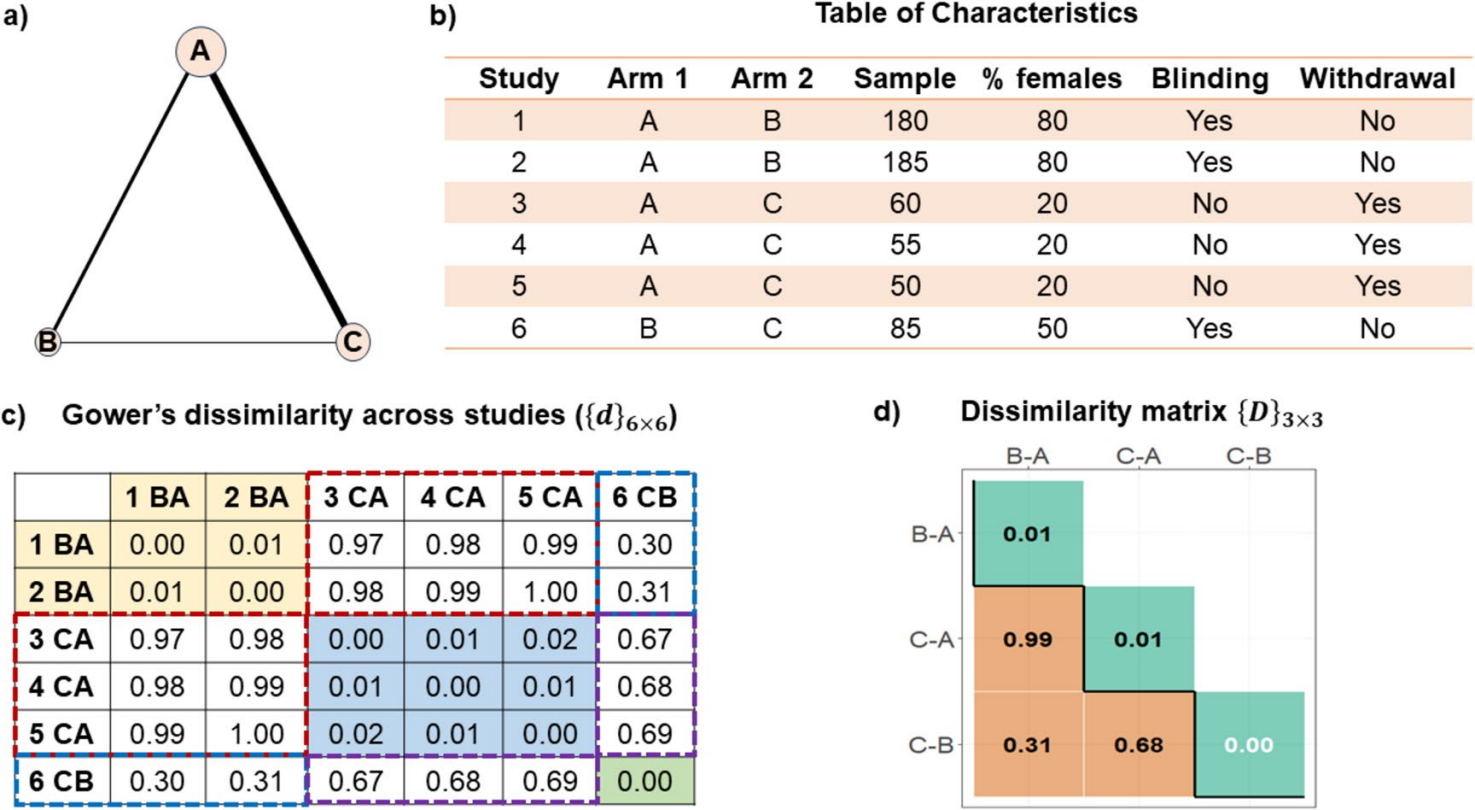
Schematic presentation of the proposed approach to transitivity evaluation using (**a**) a fictional network of three treatments and (**b**) a dataset of six studies with four fully observed characteristics considered important effect modifiers. (**c**) The proposed approach yields a symmetric matrix with Gower’s dissimilarities for all study pairs and (**d**) a square matrix with dissimilarities within each comparison (diagonal) and between two comparisons (off-diagonals)

To showcase the new approach to transitivity evaluation [27], I consider a fictional connected network with three treatments informed by six two-arm studies ($\:N=6$) (Fig. 1a) and a table with four (study-level aggregate) characteristics (Fig. 1b). The network has three (observed treatment) comparisons: BA, CA, and CB. For simplicity, I assumed that the studies reported all four characteristics; in practice, missing a characteristic in at least one study is common in published systematic reviews. Additional file 2: Methods B describes the strategy for handling missing cases when applying our proposed approach [35].

#### Step 1: Dissimilarity matrix of all study pairs

We consider the Gower dissimilarity (GD) coefficient to construct the dissimilarity matrix for all pairs of studies in the network based on a mix of numeric and non-numeric characteristics, which is the case here (Fig. 1b) [36, 37]. It takes values from 0 (complete similarity) to 1 (complete dissimilarity) and is calculated using a two-step approach. First, for the numeric characteristics, we use the following formula that essentially yields a normalised value [36]:

$$\:{d\left(x,y\right)}_{i}=\frac{\left|{x}_{i}-{y}_{i}\right|}{{R}_{i}}$$

where $\:{d\left(x,y\right)}_{i}$ is the dissimilarity between studies $\:x$ and $\:y$ for characteristic $\:i$, $\:{x}_{i}$ and $\:{y}_{i}$ refer to the values of that characteristic in the two studies and $\:{R}_{i}$ is the range for that characteristic. It holds that $\:{d\left(x,y\right)}_{i}=0$, when $\:{x}_{i}={y}_{i}$; otherwise, $\:{d\left(x,y\right)}_{i}\in\:\left(\left.\text{0,1}\right]\right.$. For the binary characteristics, we use the following formula [36]:

$$\:{d\left(x,y\right)}_i=\left\{\begin{array}{lc}1,&{\text{if}}\;x_i\neq y_i\\0,&{\text{if}}\;x_i=y_i\end{array}\right.$$

Then, for each study pair, we pool the dissimilarities across the four characteristics to obtain the corresponding GD value using the weighted arithmetic mean [36]:

$$\:d\left(x,y\right)=\frac{\sum\:_{i=1}^{4}{{\delta}_{xy,i}d\left(x,y\right)}_{i}}{\sum\:_{i=1}^{4}{\delta}_{xy,i}}$$

with $\:{\delta}_{xy,i}$ being the weight defined as

$$\delta_{xy,i}=\left\{\begin{array}{l}0,\;\mathrm{if}\;x_1\ \mathrm{or}\;y_1\;\mathrm{is}\;\mathrm{missing}\\1,\;\mathrm{if}\;\;x_{\mathit1}\ \mathrm{and}\;y_{\mathit1}\mathit\;\mathrm{are}\;\mathrm{observed}\end{array}\right.$$

Since all studies report all characteristics, $\:{\delta\:}_{xy,i}=1,$ and the GD formula coincides with the simple arithmetic mean. Figure 1c presents the symmetric GD matrix, $\:{\left\{d\right\}}_{6\times\:6}$, for all pairs among the six studies.

#### Step 2: Overall dissimilarity within and between comparisons

We turn the symmetric submatrices (coloured cells) of the GD matrix $\:{\left\{d\right\}}_{6\times\:6}$ (Fig. 1c) into a compact measurement that quantifies the overall dissimilarity *within* the corresponding comparison, referred to as *within-comparison dissimilarity*, using the root mean square of the non-diagonal elements in the corresponding submatrices of $\:{\left\{d\right\}}_{6\times\:6}$ [27]:

$$\:{D}_{p}^{W}=\sqrt{\frac{\sum_{j}{{d\left(x,y\right)}_{\left(j\right)}}^{2}}{\left(\genfrac{}{}{0pt}{}{k}{2}\right)}}$$

with $\:{d\left(x,y\right)}_{\left(j\right)}$ being the GD value $\:j=\text{1,2},\dots\:,\left(\genfrac{}{}{0pt}{}{k}{2}\right)$ between two studies of comparison $\:p$. This formula coincides with the formula for the population standard deviation after replacing the mean with zero. We preferred the root mean square to the arithmetic mean to gauge the spread of the dissimilarities from zero, where zero implies complete similarity [27]. In our example, the within-comparison dissimilarities ($\:{D}^{W}$) are

$$\:{D}_{BA}^{W}=\sqrt{\frac{{0.01}^{2}}{1}}=0.01$$

$$\:{D}_{CA}^{W}=\sqrt{\frac{{0.01}^{2}+{0.02}^{2}+{0.01}^{2}}{\left(\genfrac{}{}{0pt}{}{3}{2}\right)}}=0.01$$

for treatment comparisons BA and CA, respectively. CB is a single-study comparison; hence, dissimilarity does not apply.

Using the root mean square of all elements in the submatrices of $\:{\left\{d\right\}}_{6\times\:6}$ referring to inter-comparisons (coloured dotted frames) (Fig. 1c), we obtain the overall dissimilarity *between* comparisons ($\:{D}^{B}$), referred to as *between-comparison dissimilarity* [27]:

$$\:{D}_{CA,BA}^{B}=\sqrt{\frac{{0.97}^{2}+{0.98}^{2}{+\:0.99}^{2}+{0.98}^{2}+{0.99}^{2}+{1.00}^{2}}{6}}=0.99$$

$$\:{D}_{CA,CB}^{B}=\sqrt{\frac{{0.67}^{2}+{0.68}^{2}{+\:0.69}^{2}}{3}}=0.68$$

$$\:{D}_{CB,BA}^{B}=\sqrt{\frac{{0.30}^{2}+{0.31}^{2}}{2}}=0.31$$

for CA versus BA, CA versus CB, and CB versus BA, respectively. We arrange the within-comparison dissimilarities in the main diagonal and the between-comparison dissimilarities in the off-diagonals of the overall dissimilarity matrix $\:{\left\{D\right\}}_{3\times\:3}$ (Fig. 1d), with the comparisons appearing on the columns and rows of $\:{\left\{D\right\}}_{3\times\:3}$.

The elements of $\:{\left\{D\right\}}_{3\times\:3}$ are on the same scale as the GD metric. Equivalently, $\:{D}^{W}$ and $\:{D}^{B}$ indicate approximately the percentage of mismatched study pairs for at least one characteristic within a comparison and between two comparisons, respectively, allowing $\:{\left\{D\right\}}_{3\times\:3}$ to also be interpreted in the percentage scale. This realisation can facilitate specifying sensible thresholds to interpret the extent of overall dissimilarity within and between comparisons.

#### Step 3: Thresholds of low dissimilarity

The $\:D$ value ($\:{D}^{w}$ or $\:{D}^{B}$) expresses the average dispersion of the corresponding dissimilarities from zero, with dissimilarities measured in the interval $\:\left[0,\:1\right]$. Implicitly, $\:{D}^{w}$ and $\:{D}^{B}$ measure variability within and between comparisons in the per cent scale. A similar metric, to some extent, is the $\:{I}^{2}$ statistic, which measures the percentage of total variability due to between-study heterogeneity in a meta-analysis and, hence, it is bounded in the interval $\:\left[0,\:1\right]$ [38]. By virtue of their different definitions, the values of $\:{D}^{w}$ and $\:{D}^{B}$ cannot be mapped one-to-one to the $\:{I}^{2}$ values; however, we can adopt the $\:{I}^{2}$ values strictly based on their indication that lower values reflect low inconsistency in the study results; otherwise, material inconsistency. Hence, lower $\:{D}^{w}$ and $\:{D}^{B}$ values would indicate likely low within-comparison and between-comparison dissimilarity; lower between-comparison dissimilarity may indicate likely transitivity.

There is currently no empirical study on the extent of overall dissimilarity in different design settings. Therefore, we adopted the predictive distributions for the $\:{I}^{2}$ statistic in a future meta-analysis with a mixed outcome as developed by Rhodes et al. [39] to define the threshold of low dissimilarity [27]. The authors proposed different predictive distributions depending on different outcome and treatment-comparator types and the average sample size of the included studies (Tables 3 and 8 in [39]).

We may use the median of the selected predictive distribution for the $\:{I}^{2}$ statistic as a threshold of low dissimilarity, and define low dissimilarity as a $\:D$ value ($\:{D}^{w}$ or $\:{D}^{B}$) below the median of the predictive distribution; otherwise, the dissimilarity may be ‘likely concerning’ [27]. Alternatively, the third quartile may also be considered as a low dissimilarity threshold, if deemed appropriate, potentially leading to more frequent signals of low dissimilarity than using the median, especially for subjective outcomes. We used these thresholds (the median and third quartile) in the context of the present empirical study to understand the commonness of (in) transitivity in different design settings and overall based on the $\:{D}^{B}$ values. In the Discussion section, I offer my position about the routine use of these thresholds outside the empirical study purpose.

**Fig. 2:**
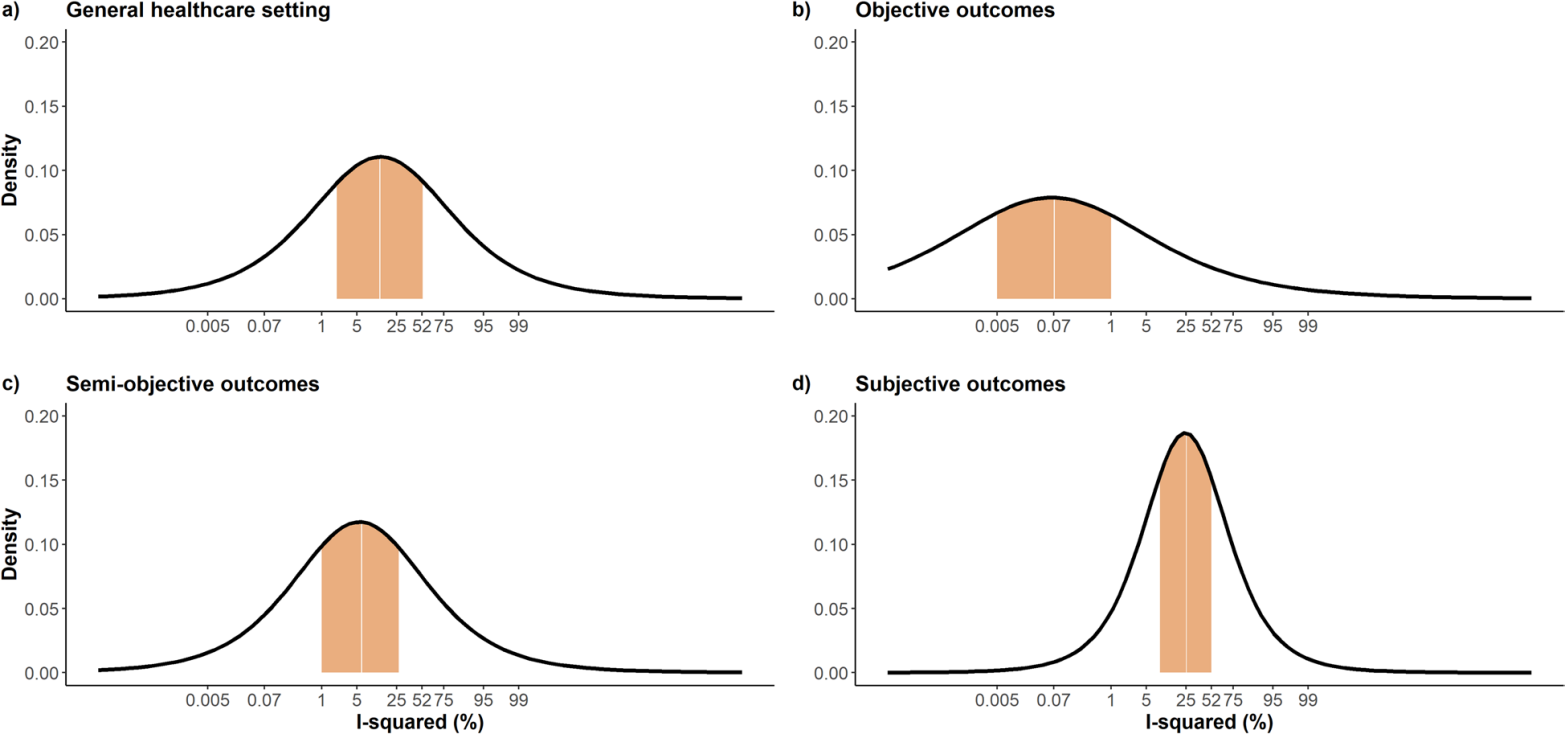
Density plots on the predictive distribution of $\:{I}^{2}$ for a future placebo-controlled meta-analysis with moderately sized studies (between 50 and 200 participants) on (**a**) general healthcare setting, (**b**) objective outcome, (**c**) semi-objective outcome, and (**d**) subjective outcome. The orange area indicates the interquartile range of $\:{I}^{2}$ values for each distribution. The median value is highlighted in white. The density plots have been created based on the information from Tables 3 and 8 for mixed outcomes in Rhodes et al. [39]

Table 1 presents different dissimilarity thresholds for a general healthcare setting and the design factors above. For instance, Fig. 2 illustrates the predictive distribution of $\:{I}^{2}$ for the general healthcare setting and the three outcome types in a placebo-controlled meta-analysis. Note the very low quartile $\:{I}^{2}$ values for the objective outcomes compared to the other settings, with subjective outcomes exhibiting the largest quartile values.

**Table 1:** Thresholds of low dissimilarity for several design factors^a^

Design factor	Pharmacological versus placebo/control		Pharmacological versus pharmacological	Any non-pharmacological
General health setting	0.13 (0.53)			
Average study size below 50 participants				
All-cause mortality	0.0007^b^ (0.01)^c^		0.0004 (0.005)	0.0007 (0.01)
Semi-objective	0.06 (0.28)		0.04 (0.16)	0.06 (0.29)
Subjective	0.25 (0.53)		0.16 (0.32)	0.24 (0.55)
Average study size between 50 and 200 participants				
All-cause mortality	0.0007 (0.01)		0.0004 (0.005)	0.0007 (0.01)
Semi-objective	0.06 (0.27)		0.04 (0.16)	0.06 (0.29)
Subjective	0.25 (0.52)		0.16 (0.32)	0.23 (0.55)
Average study size over 200 participants				
All-cause mortality	0.0008 (0.01)		0.0005 (0.006)	0.0007 (0.01)
Semi-objective	0.07 (0.30)		0.04 (0.18)	0.06 (0.33)
Subjective	0.28 (0.56)		0.18 (0.35)	0.26 (0.58)

^a^The content results from Tables 3 and 8 in [39] and refers to the median and third quartile of the predictive distribution for $\:{I}^{2}$ in a future meta-analysis with mixed outcome data. We considered the thresholds of all-cause mortality for the objective outcomes

^b^The numbers are not in per cent

^c^The numbers outside and within the parentheses refer to the median and third quartile, respectively, of the corresponding predictive distribution for $\:{I}^{2}$

Using the dissimilarity threshold for a general healthcare setting (median equals 0.13), there was low intra-comparison dissimilarity for BA and CA (Fig. 1d). Dissimilarity was ‘likely concerning’ for all inter-comparisons, with $\:{D}_{CA,BA}^{B}$ being almost one since the BA and CA studies had completely different settings (Fig. 1b). Therefore, there is evidence of likely intransitivity in the network: a CB indirect effect based on the BA and CA studies would likely be biased.

### Data analysis

#### Description of the database

Initially, the database was outlined using the median, interquartile range (IQR) and range to summarise the number of studies, treatments and observed comparisons across the datasets. The same descriptive statistics were used to summarise characteristics pertinent to transitivity evaluation: numeric and non-numeric extracted characteristics, dropped characteristics due to missing cases and percentage of missing cases across the datasets.

#### Investigating overall dissimilarity and commonness of likely intransitivity

The distribution of GD ($\:{\left\{d\right\}}_{N\times\:N}$), $\:{D}^{w}$ and $\:{D}^{B}$ was illustrated per outcome and treatment-comparator types using violin plots with integrated interval bars. I calculated the proportion of comparisons and pairs of comparisons with low and ‘likely concerning’ dissimilarity using thresholds tailored to the design factors of each dataset (Table 1). Transitivity was likely questionable in datasets with at least one inter-comparison of ‘likely concerning’ dissimilarity (i.e., based on the $\:{D}^{B}$ values); thus, the percentage of such datasets was calculated. Only datasets containing at least four extracted characteristics were analysed, effectively an abstract threshold, to obtain meaningful results from our approach; this led to a further reduction of the analysed datasets, as revealed in the Results section. Results were presented for two thresholds of low dissimilarity (median and third quartile) using grouped bar plots, with the x-axis referring to the thresholds and the different colours referring to the transitivity conclusion (likely present or likely absent) based on the $\:{D}^{B}$ values.

#### Demonstrating the disadvantages of multiple statistical tests for transitivity assessment

For each dataset, I used the characteristics exactly as extracted and conducted as many statistical tests as the number of characteristics. Specifically, the F-test of the one-way ANOVA was employed for the numerical characteristics and the chi-squared test for the categorical characteristics. For each statistical test in each dataset, the independent variable was the observed comparisons, and the dependent variable was the characteristic. For datasets containing at least one comparison informed by a single study, the F-test would not perform. Hence, these numeric characteristics would be removed from the corresponding datasets. I also expected neither test to perform for characteristics with the same value in all studies. Therefore, these characteristics would also be removed from the corresponding datasets. Lastly, the p-value of the F-test cannot be defined for characteristics that have the same values in all studies of at least one comparison informed by more than one study. In such a scenario, the chi-squared test would not be credible for having small expected values. I recorded how often the issues mentioned above occurred as they would indicate the limitations of using multiple statistical tests for transitivity assessment.

#### Investigating the conclusions from multiple statistical tests and the approach of study dissimilarities

Before applying the statistical tests, the datasets were pre-processed by removing characteristics that hindered either statistical test, as described above. The final set included only datasets with at least four characteristics. The multiple statistical tests were conducted at a significance level of 5%. For each statistical test, the null hypothesis indicated no association between the observed comparisons (independent variable) and the characteristic (dependent variable). Then, the alternative hypothesis indicated an association between the observed comparisons and the characteristic. When the two-sided p-value was below 5% for at least one characteristic, the network corresponding to that dataset was deemed likely intransitive (conclusive evidence); otherwise, the evidence was inconclusive. The approach of study dissimilarities was applied to those datasets considered in multiple statistical tests, using two thresholds of low dissimilarity (median and third quartile) to conclude whether transitivity is likely or questionable based on the $\:{D}^{B}$ values. Then, I calculated the number and proportion of datasets with the same or different conclusions regarding likely intransitivity under the approach of study dissimilarities (based on the $\:{D}^{B}$ values) and multiple statistical tests. The results were illustrated using a stacked bar plot, with the x-axis referring to conclusive (i.e., likely intransitivity) and inconclusive evidence under multiple statistical tests and the different bar colours referring to likely transitivity or intransitivity based on the approach of study dissimilarities. Results were presented separately for the two thresholds of low dissimilarity. I also calculated the family-wise error rate for each analysed dataset to demonstrate the extent of imminent multiplicity due to multiple tests in each dataset.

All analyses were performed using the statistical software R, version 4.3.3 [40]. The *rnmamod* R package was used to apply the approach of study dissimilarities [27] and the *ggplot2* R package to create the figures [41, 42]. The functions *oneway.test* and *chisq.test* were employed to conduct the F-test of the one-way ANOVA and the chi-squared test, respectively. The data that support the findings of the present study can be found in the *tracenma* R package [28]. Additional file 2: Methods C provides an example of how to use the *tracenma* and *rnmamod* R packages. The present empirical study has not been registered, nor is a protocol available.

## Results

### Description of the database

The 214 datasets had a median of 17 studies (range: 5–145) and seven treatments (range: 3–40) (Table 2). There were three datasets with all treatments being compared. The remaining datasets contained 7–90% (median: 40%) of the possible pairwise comparisons in the corresponding networks. Most datasets had at least one single-study comparison (86%; $\:n=183$), ranging from 7 to 100% of the observed comparisons (median: 44%), and considered objective outcomes (42%; $\:n=89$), followed by semi-objective ones (38%; $\:n=81$) (Additional file 3: Fig. S1). Comparisons of pharmacological treatments with placebo dominated all outcomes (Additional file 3: Fig. S1).

**Table 2:** Characteristics related to network and transitivity evaluation

**Characteristic**	**Median**	**IQR**	**Range**
*Network related*			
Number of studies	17	(11, 29)	(5, 145)
Number of treatments^a^	7	(5, 9)	(3, 40)
Observed comparisons^b^ (%)	42	(32, 53)	(7, 100)
Single-study comparisons^c^ (%)	44	(29, 60)	(7, 100)
*Transitivity evaluation related*			
Number of extracted characteristics^d^	12	(8, 15)	(4, 41)
Numeric^e^ (%)	55	(33, 80)	(0, 100)
Non-numeric^e^ (%)	45	(20, 67)	(0, 100)
Total missing data^f^ (%)	7.1	(2.6, 12.9)	(0.2, 32.9)
Missing data per characteristic^f^ (%)	20.0	(8.2, 39.2)	(0.6, 97.6)
Characteristics with missing data^f^ (%)	33.3	(20.0, 50.0)	(5.3, 93.8)
Dropped characteristics^g^ (%)	3.0	(1, 5)	(1, 16)

IQR, interquartile range

^a^There was no outcome data in three studies of one network with four treatments. These studies would not be considered in the network meta-analysis; hence, they were excluded from the corresponding dataset, leading to one treatment less, without compromising network connectivity

^b^There were three fully connected networks; after excluding these networks, the percentage median dropped to 40 (% range: 7–90)

^c^Of the networks with at least one single-study comparison, one network included only single-study comparisons

^d^Some extracted numeric characteristics were summarised using two descriptive statistics, such as mean and standard deviation or minimum and maximum, and they were accounted for in the number of extracted characteristics

^e^One network dataset included only non-numeric extracted characteristics, and 27 included only numeric extracted characteristics

^f^Based on 177 network datasets with at least one missing case

^g^Based on the 124 network datasets that ‘lost’ at least one extracted characteristic

The extracted characteristics varied greatly across the datasets, ranging from 4 to 41 (median: 12) (Table 2 and Additional file 3: Fig. S2a). Most extracted characteristics were numeric (median: 55% versus 45% for numeric versus non-numeric). There was at least one missing case in most datasets (83%; $\:n=177$), ranging from 0.2 to 32.9% (median: 7.1%). In these datasets, the missing cases per characteristic ranged from 0.6 to 97.6% (median: 20%), and the characteristics with at least one missing case ranged from 5.3 to 93.8% (median: 33.3%). Most datasets (58%; $\:n=124$) contained at least one characteristic missing in almost all studies of at least one non-single-study comparison. These characteristics ranged from 1 to 16% (median: 3%) and were dropped from the corresponding datasets. After dropping these characteristics, five datasets contained less than four characteristics and were removed from the subsequent analyses: the median of characteristics decreased to 10 (IQR: 7–13, range: 4–35) (Additional file 3: Fig. S2b). A total of 209 datasets were considered for the subsequent analyses.

### Investigating overall dissimilarity and commonness of intransitivity

#### Gower dissimilarity coefficient

The median GD was 0.30 (IQR: 0.19–0.42) across the datasets, covering all possible values (range: 0.00–1.00). A quite similar extent of the GD values was observed for the different outcome and treatment-comparator types (range of medians: 0.26–0.40), with the upper bounds of the IQR showcasing some notable study dissimilarity across the datasets (Additional file 1: Table S2 and Additional file 3: Fig. S3). Seventy-three (35%) datasets had at least one zero GD value. Within these datasets, zero GD values were less common than the non-zero GD values, as the zero GD values only ranged from 0.09 to 14.3% (median: 1.06%, IQR: 0.46–2.93%). The commonness of non-zero GD values indicated that dissimilarity among studies governed the datasets, regardless of outcome and treatment-comparator type (Additional file 1: Table S3).

#### Within-comparison dissimilarity

Based on non-single-study comparisons, $\:{D}^{W}$ ranged from 0.00 to 0.93 (median: 0.30), exhibiting material within-comparison dissimilarity in the middle 50% of the comparisons (IQR: 0.21–0.39). Overall, the distribution of $\:{D}^{W}$ had a similar extent when considering the different outcomes and treatment-comparator types (Additional file 1: Table S4 and Additional file 3: Fig. S4).

Under the median for low dissimilarity threshold, all comparisons relating to objective outcomes exhibited ‘likely concerning’ within-comparison dissimilarity (Additional file 3: Fig. S5) for having an almost zero dissimilarity threshold (Table 1), making it difficult to detect comparisons with low $\:{D}^{W}$ values. With semi-objective outcomes, few comparisons had low dissimilarity (1.8–9.1%). Low within-comparison dissimilarity was more common with subjective outcomes (Additional file 3: Fig. S5) for having higher dissimilarity quartiles (Table 1). Using the third quartile, and thus, higher thresholds of low dissimilarity, led to more comparisons with low dissimilarity for semi-objective (18.2–50.4%) and almost all comparisons for subjective outcomes (93.6% and 95%) (Additional file 3: Fig. S5).

#### Between-comparison dissimilarity and transitivity commonness

Notable dissimilarity was also observed among most comparisons (IQR: 0.23–0.42), with a median $\:{D}^{B}$ of 0.33 and covering all possible values (range: 0.00–1.00). Overall, a similar extent of $\:{D}^{B}$ was observed in all combinations of outcome and treatment-comparator types (Table 3), demonstrating the ubiquity of study dissimilarity across comparisons. Additional file 3: Fig. S6 illustrates the density of $\:{D}^{B}$ for different outcome and treatment-comparator types.

**Table 3:** Summary of between-comparison dissimilarities from 209 datasets

**Outcome**	**Treatment-comparator**	**Median**	**IQR**	**Range**	**Pairs comp**^**a**^
Objective	Pharmacol. versus Placebo	0.29	0.22 – 0.37	0.00 – 0.77	3 – 2080
	Pharmacol. versus Pharmacol.	0.31	0.21 – 0.41	0.00 – 0.83	3 – 435
	Non-pharmacol. versus Any	0.30	0.23 – 0.38	0.00 – 0.93	3 – 231
Semi-objective	Pharmacol. versus Placebo	0.33	0.24 – 0.42	0.00 – 1.00	3 – 1596
	Pharmacol. versus Pharmacol.	0.31	0.05 – 0.52	0.00 – 0.80	6 – 36
	Non-pharmacol. versus Any	0.41	0.32 – 0.50	0.00 – 0.96	10 – 561
Subjective	Pharmacol. versus Placebo	0.32	0.23 – 0.41	0.00 – 0.90	3 – 2628
	Pharmacol. versus Pharmacol.	0.38	0.28 – 0.44	0.12 – 0.56	15
	Non-pharmacol. versus Any	0.40	0.33 – 0.46	0.00 – 0.68	6 – 378

*IQR* Interquartile range, *Pairs comp* Pairs of comparisons, *Pharmacol* Pharmacological

^a^Range of pairs of comparisons across the corresponding datasets

Like with $\:{D}^{W}$, low dissimilarity was difficult to detect for objective outcomes using the median as low dissimilarity threshold (0.6–1.5% pairs of comparisons) (Additional file 3: Fig. S7). Contrariwise, slightly more pairs of comparisons were associated with low dissimilarity for semi-objective outcomes (0.7–17.5%) and notably more for subjective outcomes (6.7–35.8%). When using the third quartile, the pairs of comparisons with low dissimilarity increased further for semi-objective (20.3–45%) and subjective outcomes (46.7–94.6%).

**Fig. 3:**
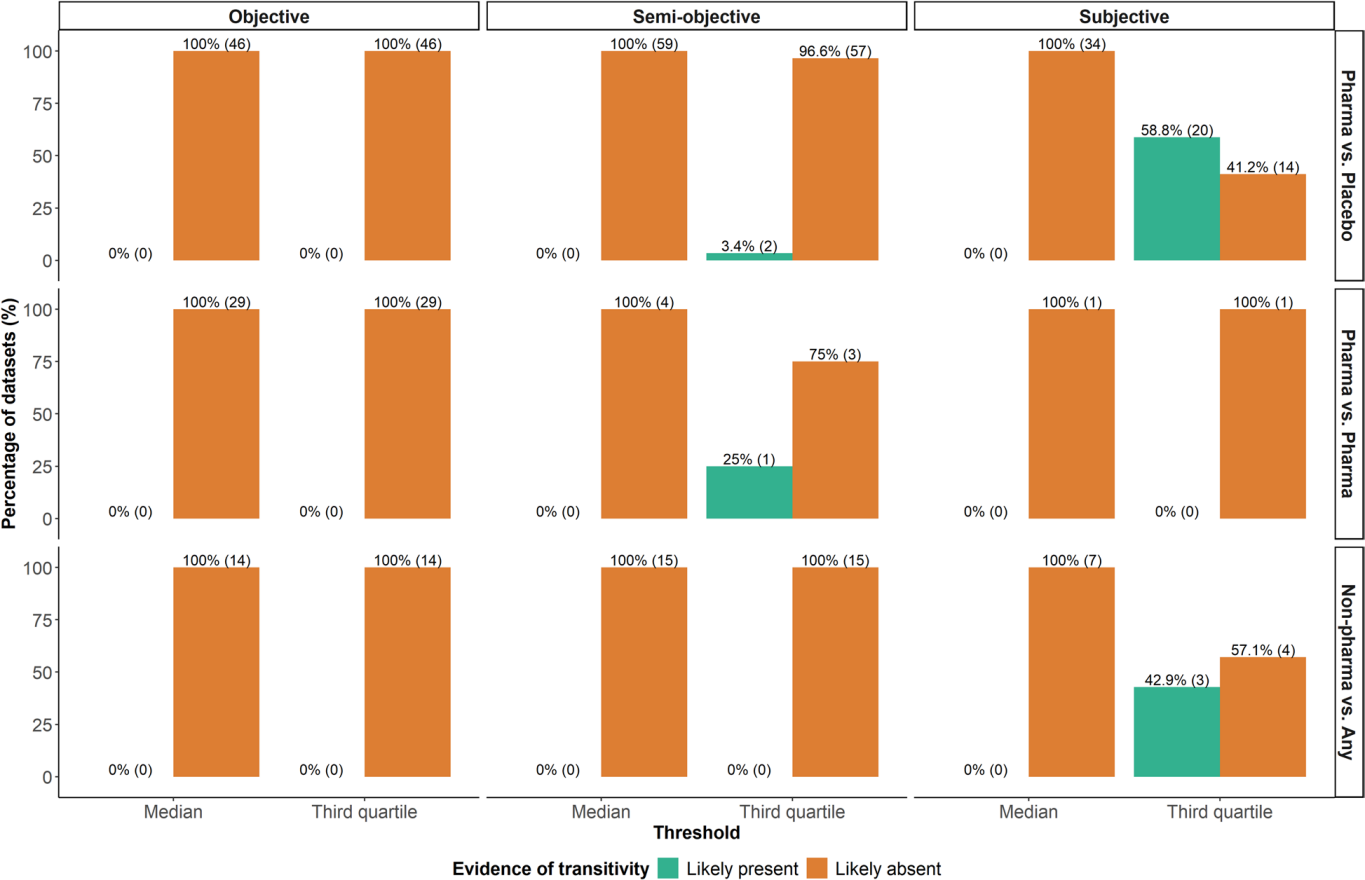
Grouped bar plots on the percentage of datasets with ‘likely present’ (green bars) and ‘likely absent’ (orange bars) transitivity based on two thresholds of low dissimilarity: the median and third quartile of the selected predictive distribution for $\:{I}^{2}$. The thresholds are tailored to the outcome (objective, semi-objective, and subjective) and treatment-comparator types (pharmacological versus placebo/control, pharmacological versus pharmacological, and non-pharmacological versus any). The numbers in parentheses refer to the number of corresponding datasets. Pharma, pharmacological

**Fig. 4:**
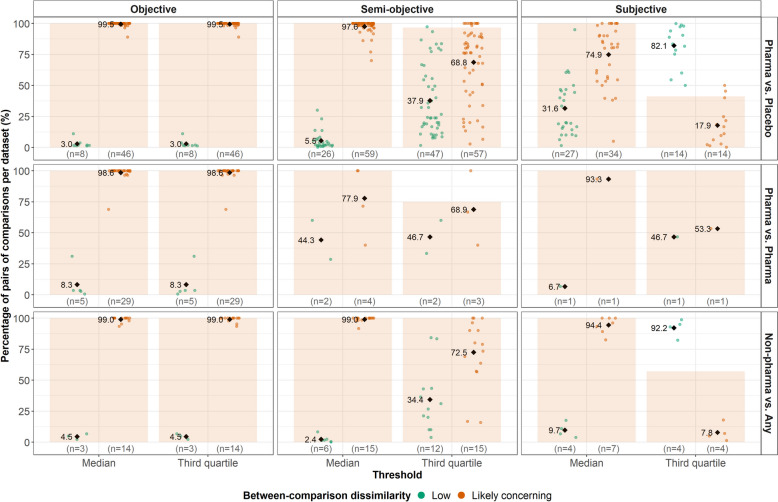
Dot plots on the percentage of pairs of comparisons with ‘low’ (green dots) and ‘likely concerning’ (orange dots) between-comparison dissimilarity for datasets with likely *intransitivity*. Two thresholds of low dissimilarity have been considered: the median and third quartile of the selected predictive distribution for $\:{I}^{2}$. The thresholds are tailored to the outcome (objective, semi-objective, and subjective) and treatment-comparator types (pharmacological versus placebo/control, pharmacological versus pharmacological, and non-pharmacological versus any). The background bars refer to the percentage of datasets with likely *intransitivity*. The numbers in parentheses refer to the number of datasets. The black diamonds refer to the average percentage of pairs of comparisons. Pharma, pharmacological

Transitivity was likely absent in all datasets under the median as a threshold for $\:{D}^{B}$ (Fig. 3). The third quartile revealed transitivity to be likely present in three and 23 datasets for semi-objective and subjective outcomes, respectively, highlighting the commonness of intransitivity in the database even for larger thresholds of low dissimilarity (Fig. 3). Restricting to datasets with likely absent transitivity shed light on the frequency of low and ‘likely concerning’ $\:{D}^{B}$ values (Fig. 4). Specifically, for objective outcomes, almost all pairs of comparisons had ‘likely concerning’ $\:{D}^{B}$ values regardless of threshold. Using the third quartile as a threshold led to a decrease in the average percentage pairs of comparisons with ‘likely concerning’ $\:{D}^{B}$ values for semi-objective outcomes and even more for subjective outcomes.

### Demonstrating the disadvantages of multiple statistical tests for transitivity assessment

Of the 209 analysed datasets, there were 178 with at least one single-study comparison. After removing the numeric characteristics from these datasets because one-way ANOVA did not perform, 67 (32%) datasets were excluded for having less than four characteristics. Subsequently, 115 characteristics (median: 1, range: 1–8) containing the same value across all studies were dropped from 60 datasets, further excluding 15 datasets for having less than four characteristics. Consequently, only 127 (61%) datasets were considered suitable for statistical testing. The chi-squared approximation was questionable (expected frequencies were below 5) for all characteristics in 119 datasets. The F-test p-value was undefined for 35 numeric characteristics (median: 1, range: 1–5) in 21 (16%) datasets for having the same value in at least one non-single-study comparison. After dropping these characteristics from the corresponding datasets, the analysed characteristics ranged from 25 to 96% (median: 88%, IQR: 67–91%).

### Investigating the conclusions from multiple statistical tests and the approach of study dissimilarities

One hundred twenty-seven datasets were considered to compare the conclusions from multiple statistical tests with those from the approach of study dissimilarities. For a significance level of 5%, the family-wise error rate ranged from 5 to 69% (median: 30%) across the 127 analysed datasets ascribed to multiple testing.

**Fig. 5:**
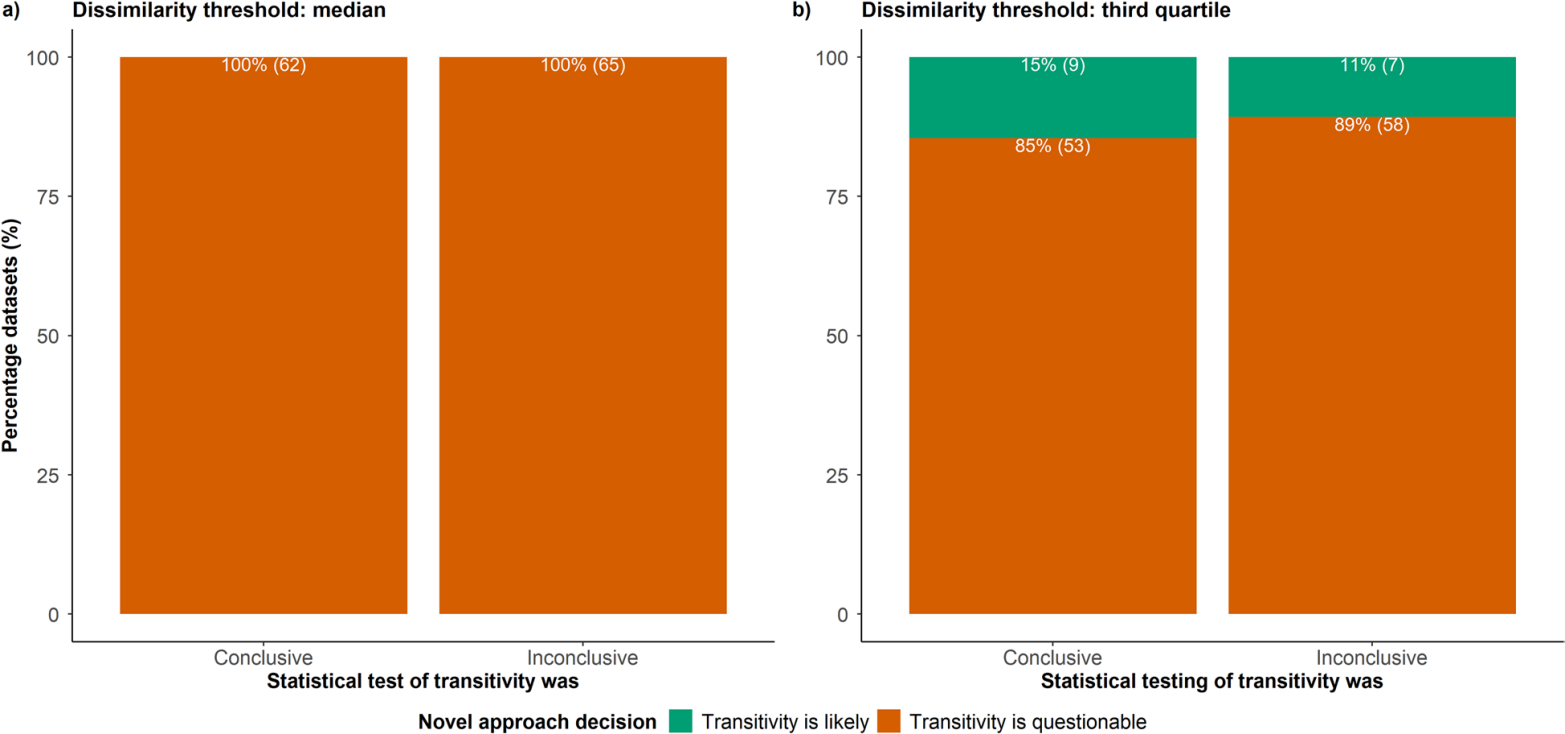
Stacked bar plots on the conclusions about the plausibility of transitivity based on the study dissimilarity approach to transitivity assessment and statistical testing in 127 datasets. The approach of study dissimilarities was employed using two thresholds of low dissimilarity for the between-comparison dissimilarities: the median and third quartile of the selected predictive distribution for $\:{I}^{2}$

Statistical testing indicated 62 (49%) datasets as likely intransitive for containing at least one statistical test with a p-value below 5% and, hence, conclusive evidence (Fig. 5). The evidence was inconclusive for the remaining 65 (51%) datasets (Fig. 5), hindering conclusions about the plausibility of transitivity. On the contrary, the approach of study dissimilarities indicated all 127 datasets to have ‘likely concerning’ dissimilarity, and hence, likely intransitivity, for all pairs of treatment comparisons based on the median as low dissimilarity threshold (Fig. 5a). Under the third quartile, the approach of study dissimilarities concurred with statistical testing that transitivity may be questionable in 53 of these 62 (85%) datasets (Fig. 5b). Of the 65 datasets with inconclusive statistical testing, the approach of study dissimilarities indicated 58 (89%) datasets as having likely intransitivity (Fig. 5b). Overall, statistical tests were comparable with the approach of study dissimilarities regarding conclusions of likely intransitivity. However, only the approach of study dissimilarities warranted a conclusion about transitivity’s potential plausibility when statistical tests were inconclusive.

## Discussion

Study dissimilarity attributed to variations in the distribution of effect modifiers across studies should be expected. The present empirical study revealed the ubiquity of study dissimilarity within and across comparisons, regardless of design setting, based on study dissimilarities concerning the effect modifiers’ distribution. Using empirically-driven dissimilarity thresholds, ‘likely concerning’ between-comparison dissimilarity, manifesting as likely intransitivity, was common in NMA, necessitating scrutiny of the evidence base.

Dissimilarity thresholds aid in interpreting the extent of overall dissimilarity within and between the observed comparisons. Particularly thresholds for the $\:{D}^{B}$ values may also guide decisions about the statistical analysis, such as conducting NMA on the whole evidence base, splitting the network into transitive subnetworks, or refraining from NMA. Therefore, defining the dissimilarity thresholds is crucial and should be tailored to the constitution of the investigated population, treatments and outcomes. Content expertise alongside relevant empirical evidence should drive the definition of dissimilarity thresholds. Developing dissimilarity thresholds customised to the investigated healthcare field and design characteristics (e.g., outcome and treatment-comparator type) deserves investigation.

I used the predictive distributions of the $\:{I}^{2}$ statistic developed by Rhodes et al. [39] as a surrogate to define tailored thresholds of low overall dissimilarity; however, the relevance and applicability of these thresholds for the intended design settings are unknown. Therefore, the analysts are advised to use these thresholds critically when applying our approach and, when necessary, devise appropriate thresholds for the investigated research questions.

There was discordance between statistical testing and the approach of study dissimilarities for transitivity evaluation concerning the number of analysed datasets and characteristics. Thirty-nine per cent fewer datasets were used for statistical testing, corresponding to 127 datasets as opposed to 209 datasets used in our approach, with 97% of the analysed datasets losing 8–82% of the original characteristics (median: 42%) due to testing-related issues. Furthermore, there was evidence of material multiplicity due to multiple tests in each dataset, with the family-wise error ranging from 5 to 69% (median: 30%) across the 127 datasets, raising the risk of false positive conclusions. Lastly, statistical testing yielded inconclusive results for 51% of the datasets. On the contrary, for those datasets, our approach distinguished between datasets with likely transitivity and those with likely intransitivity. These shortcomings demonstrate the unsuitability of statistical testing, currently performed for each characteristic, as a method for evaluating the transitivity assumption, showcasing the approach of study dissimilarities [27] as an essential methodological alternative for transitivity evaluation.

The *tracenma* R package [28] was created, the first database with study-level aggregate characteristics extracted from several published systematic reviews with NMA to inspire further development of methodology for transitivity evaluation that can be appraised and exemplified using real-life data. Nevertheless, due to limitations in the extracted data, the *tracenma* database should be used strictly for methodological purposes and not to gain knowledge regarding the extent of overall dissimilarity in different healthcare fields. For instance, more recent systematic reviews were not included; hence, the database may not reflect current knowledge on what constitutes important effect modifiers in various healthcare fields, potentially limiting the generalisability of the findings to more recent systematic reviews with NMA [43]. A systematic survey on the quantity and relevance of reported effect modifiers in recent systematic reviews with NMA is crucial in demystifying their quality regarding the selected effect modifiers, and the awareness of the involved authors concerning the role of effect modifiers in transitivity assessment.

Several data transformations were employed to achieve a consistent format across studies using statistical methods for quantitative characteristics [33] and subjective judgments for qualitative characteristics without consulting expert opinion, potentially compromising accuracy, which may have biased our findings to some unknown extent. Ideally, the authors of the included studies should be contacted to request missing information, as this approach would maximise accuracy in the extracted data instead of using statistical approaches that offer mere approximations. Consulting experts in the investigated research field is pivotal when transforming qualitative characteristics in text format into quantitatively analysable variables or lumping multiple categories into compact ones to ensure minimum information loss. Furthermore, the relevance and completeness of the extracted characteristics as effect modifiers from the corresponding systematic reviews are unknown and depend on the reporting transparency of the primary studies and the knowledge of the involved clinicians about the investigated research field, concerns also expressed by other authors [44, 45].

Transitivity and consistency are essentially the same assumption [11]. Transitivity is necessary to obtain a credible indirect estimate for a comparison not investigated directly in the network [11]. Consistency is the extension of transitivity, and it is required to obtain a valid mixed estimate for a comparison [11]. However, evidence of transitivity may not necessarily translate into evidence of consistency [11]. Therefore, transitivity and consistency should be routinely assessed to uncover possible violations in several parts of the networks. The study dissimilarities approach can assist in straightforward and reliable transitivity assessment contrary to multiple statistical tests. However, caution should be exercised when there are missing data, as our approach employs pairwise-like deletion as a tentative solution due to a lack of guidance on dealing with missing data in the context of transitivity assessment. Caution is also required when using methods for inconsistency assessment due to power issues and other limitations delineated elsewhere in the relevant literature [8, 11, 12].

## Conclusions

Like statistical heterogeneity, study dissimilarity attributed to variations in the effect modifiers’ distribution across the studies should be expected and properly quantified. Our simple approach to transitivity evaluation revealed the ubiquity of study dissimilarities across the treatment comparisons, manifesting as likely intransitivity across the investigated networks. Unlike our approach, statistical testing was applicable only in a handful of datasets, yielding inconclusive results in many datasets and being prone to multiplicity issues. The analysed datasets are compiled in the *tracenma* R package to help statisticians and methodologists develop further methods for evaluating the transitivity assumption, the cornerstone of NMA.

## Supplementary Information

Additional file 1: Table S1. Database for transitivity evaluation based on the nmadb database [29]. Table S2. Summary of Gower’s dissimilarity coefficient from 209 datasets. Table S3. Summary of percentage of zero Gower’s dissimilarity coefficient from 209 datasets. Table S4. Summary of within-comparison dissimilarities from 209 datasets.

Additional file 2: Methods A. Information on dataset configuration and extraction challenges. Methods B. Handing missing cases in our approach. Method C. Illustrate tracenma and rnmamod R packages with one example.

Additional file 3: Figure S1. Grouped bar plots on the distribution of the different outcome types and treatment-comparator types in 214 datasets referring to networks of treatments for the primary outcomes. Figure S2. Dot plots with integrated interval bars on the number of extracted characteristics from 214 datasets (plot a)) and the reduced set of 209 datasets (plot b)). Figure S3. Violin plots with integrated interval bars on the Gower's dissimilarity coefficient for each combination of outcome type with treatment-comparator type. Figure S4. Violin plots with integrated interval bars on the within-comparison dissimilarities for each combination of outcome type with treatment-comparator type. Figure S5. Grouped bar plots on the percentage of treatment comparisons with 'low' and 'likely concerning' within-comparison dissimilarity based on two thresholds of low dissimilarity. Figure S6. Violin plots with integrated interval bars on the between-comparison dissimilarities for each combination of outcome type with treatment-comparator type. Figure S7. Grouped bar plots on the percentage of pairs of treatment comparisons with 'low' and 'likely concerning' between-comparison dissimilarity based on two thresholds of low dissimilarity.

## Data Availability

The data supporting the present study’s findings can be found in the tracenma R package (https://CRAN.R-project.org/package=tracenma). The functions related to the present study are publicly available at https://github.com/LoukiaSpin/Empirical-evaluation-transitivity-commonness.git.

## Abbreviations

ANOVA: Analysis of variance
GD: Gower dissimilarity
IQR: Interquartile range
NMA: Network meta-analysis
