# Supplementary material for: An empirical study on 209 networks of treatments revealed intransitivity to be common and multiple statistical tests suboptimal to assess transitivity
Source: BMC Med Res Methodol. 2024 Dec 16;24:301. doi: 10.1186/s12874-024-02436-7 (PMC11648297; doi:10.1186/s12874-024-02436-7)
Supplement: Supplementary file 1 — Additional file 1: Table S1. Database for transitivity evaluation based on the nmadb database [29]. Table S2. Summary of Gower’s dissimilarity coefficient from 209 datasets. Table S3. Summary of percentage of zero Gower’s dissimilarity coefficient from 209 datasets. Table S4. Summary of within-comparison dissimilarities from 209 datasets. [file 12874_2024_2436_MOESM1_ESM.docx]

**Additional file 1**

**Supplementary material for the manuscript entitled 'An empirical study on 209 networks of treatments revealed intransitivity to be common and multiple statistical tests suboptimal to assess transitivity'**

Loukia M. Spineli^1^

^1^Midwifery Research and Education Unit, Hannover Medical School, Hannover, Germany

**Table S1.** **Database for transitivity evaluation based on the *nmadb* database [29]**

| **Selection process** | **Counts (%)** |
| --- | --- |
| **Networks found in the *nmadb* database** | **453** |
| **Total excluded for the following reasons** |  |
| *Unavailable data*^a^ | 167 (37%) |
| *Less than four extractable characteristics^b^* | 33 (7%) |
| *No table of characteristics was available* | 25 (6%) |
| *Characteristics were not summarised per study* | 5 (1%) |
| *Article in Hungarian* | 1 (0.2%) |
| *Article in Chinese* | 1 (0.2%) |
| *Characteristics were provided in textual form* | 1 (0.2%) |
| *Network connectivity was compromised due to missing data^c^* | 1 (0.2%) |
| *Study names or references were not contained in nmadb^d^* | 1 (0.2%) |
| *Unresponsive corresponding author to share data* | 1 (0.2%) |
| **Total eligible from the *nmadb* database** | **217 (48%)** |
| *Excluded after removing treatment-related characteristics^e^* | 3 (1.4%) |
| **Total eligible for the present study** | **214 (47%)** |

^a^The nmadb database included a binary column with values 'True' and 'False' to indicate whether outcome data were extracted for the corresponding network (e.g., number of events and randomised participants for each intervention arm of every study).

^b^We aimed to have as many characteristics as possible to obtain meaningful results from our approach to transitivity assessment; hence, we set the *abstract* threshold of at least four characteristics.

^c^Network connectivity was compromised due to completely missing characteristics in many studies of that network, leading to their exclusion from the network.

^d^Study names or references were not contained in the nmadb database, making it difficult to identify these studies in the corresponding article.

^e^Three datasets were considered ineligible for the present study after removing treatment-related characteristics concerning the dose description of the compared arms (e.g., treatment dose, frequency, duration, interval, administration, and route) for having less than four characteristics.

**Table S2.** **Summary of Gower’s dissimilarity coefficient from 209 datasets**

| **Outcome** | **Intervention-comparator** | **Median** | **IQR** | **Range** | **Datasets** |
| --- | --- | --- | --- | --- | --- |
| Objective | Pharmacol. versus Placebo | 0.28 | 0.18 – 0.40 | 0.00 – 0.99 | 46 |
|  | Pharmacol. versus Pharmacol. | 0.27 | 0.17 – 0.39 | 0.00 – 1.00 | 29 |
|  | Non-pharmacol. versus Any | 0.26 | 0.18 – 0.39 | 0.00 – 0.93 | 14 |
| Semi-objective | Pharmacol. versus Placebo | 0.30 | 0.20 – 0.42 | 0.00 – 1.00 | 59 |
|  | Pharmacol. versus Pharmacol. | 0.40 | 0.20 – 0.60 | 0.00 – 1.00 | 4 |
|  | Non-pharmacol. versus Any | 0.38 | 0.28 – 0.50 | 0.00 – 0.98 | 15 |
| Subjective | Pharmacol. versus Placebo | 0.28 | 0.17 – 0.40 | 0.00 – 0.90 | 34 |
|  | Pharmacol. versus Pharmacol. | 0.38 | 0.28 – 0.46 | 0.12 – 0.72 | 1 |
|  | Non-pharmacol. versus Any | 0.38 | 0.22 – 0.51 | 0.00 – 0.90 | 7 |

IQR, interquartile range; Pharmacol, pharmacological

**Table S3.** **Summary of % of zero Gower’s dissimilarity coefficient from 209 datasets**

| **Outcome** | **Intervention-comparator** | **Median** | **IQR** | **Range** | **Datasets** |
| --- | --- | --- | --- | --- | --- |
| Objective | Pharmacol. versus Placebo | 1.18 | 0.61 – 3.23 | 0.26 – 5.33 | 14 (30%) |
|  | Pharmacol. versus Pharmacol. | 0.86 | 0.75 – 3.30 | 0.20 – 6.67 | 9 (31%) |
|  | Non-pharmacol. versus Any | 0.72 | 0.42 – 1.06 | 0.10 – 10.90 | 6 (43%) |
| Semi-objective | Pharmacol. versus Placebo | 1.05 | 0.32 – 1.73 | 0.09 – 13.50 | 25 (42%) |
|  | Pharmacol. versus Pharmacol. | 2.60 | 2.21 – 2.99 | 1.82 – 3.38 | 2 (50%) |
|  | Non-pharmacol. versus Any | 0.31 | 0.22 – 1.14 | 0.09 – 2.90 | 7 (47%) |
| Subjective | Pharmacol. versus Placebo | 1.61 | 0.77 – 3.29 | 0.36 – 14.3 | 19 (56%) |
|  | Pharmacol. versus Pharmacol. | NA | NA | NA | 0 |
|  | Non-pharmacol. versus Any | 1.11 | 0.59 – 3.31 | 0.35 – 5.45 | 6 (86%) |

IQR, interquartile range; NA, not applicable; Pharmacol, pharmacological

**Table S4.** **Summary of within-comparison dissimilarities from 209 datasets**

| **Outcome** | **Intervention-comparator** | **Median** | **IQR** | **Range** | **% NS comp.^a^** |
| --- | --- | --- | --- | --- | --- |
| Objective | Pharmacol. versus Placebo | 0.28 | 0.21 – 0.37 | 0.01 – 0.60 | 14 – 100 |
|  | Pharmacol. versus Pharmacol. | 0.30 | 0.21 – 0.40 | 0.01 – 0.91 | 7 – 100 |
|  | Non-pharmacol. versus Any | 0.30 | 0.25 – 0.39 | 0.03 – 0.66 | 20 – 100 |
| Semi-objective | Pharmacol. versus Placebo | 0.29 | 0.20 – 0.38 | 0.00 – 0.65 | 0^b^ – 100 |
|  | Pharmacol. versus Pharmacol. | 0.48 | 0.36 – 0.58 | 0.02 – 0.61 | 20 – 78 |
|  | Non-pharmacol. versus Any | 0.33 | 0.26 – 0.42 | 0.00 – 0.93 | 26 – 100 |
| Subjective | Pharmacol. versus Placebo | 0.27 | 0.19 – 0.38 | 0.00 – 0.67 | 20 – 100 |
|  | Pharmacol. versus Pharmacol. | 0.49 | NA | NA | 17 |
|  | Non-pharmacol. versus Any | 0.40 | 0.31 – 0.45 | 0.06 – 0.57 | 20 – 88 |

IQR, interquartile range; NA, not applicable; NS comp, non-single-study comparisons; Pharmacol, pharmacological

^a^Range of percentage non-single-study comparisons across the corresponding datasets.

^b^One dataset had only single-study comparisons.
