# Supplementary material for: An empirical study on 209 networks of treatments revealed intransitivity to be common and multiple statistical tests suboptimal to assess transitivity
Source: BMC Med Res Methodol. 2024 Dec 16;24:301. doi: 10.1186/s12874-024-02436-7 (PMC11648297; doi:10.1186/s12874-024-02436-7)
Supplement: Supplementary file 2 — Additional file 2: Methods A. Information on dataset configuration and extraction challenges. Methods B. Handing missing cases in our approach. Method C. Illustrate tracenma and rnmamod R packages with one example. [file 12874_2024_2436_MOESM2_ESM.docx]

**Additional file 2**

**Supplementary material for the manuscript entitled 'An empirical study on 209 networks of treatments revealed intransitivity to be common and multiple statistical tests suboptimal to assess transitivity'**

Loukia M. Spineli^1^

^1^Midwifery Research and Education Unit, Hannover Medical School, Hannover, Germany

**Methods A: Information on dataset configuration and extraction challenges**

***Extracting study-level aggregate clinical and methodological characteristics***

For each eligible network from the *nmadb* database [29], we retrieved the main article and supplementary material (if available) and sought tables summarising clinical and methodological characteristics at the study level. We considered the study names (or references) and treatment comparisons found in the *nmadb* database [29] to help locate the studies in the corresponding articles (and supplementary material) and extract the characteristics available in the relevant table(s). Hence, we have created one dataset for each eligible network containing extracted study-level aggregate, clinical and methodological characteristics.

Each dataset in the *tracenma* database [28] has a long format, with two-arm studies occupying one row and multi-arm studies occupying as many rows as the pairwise comparisons among the investigated treatments. Each study-level characteristic occupies one column in the dataset, with characteristics reported per treatment arm and pertaining to treatment features (e.g., dose, dosage, and route) occupying one column for the experimental and another for the control arm in the corresponding comparison. The present study did not consider treatment-related characteristics, as explained in the subsequent section. Quantitative characteristics summarised in each study using two descriptive statistics, such as mean with standard deviation or minimum with maximum, occupied two columns in the dataset.

Clinical characteristics included study-level aggregate information on the demographics (e.g., percentage female, mean and standard deviation of age), the investigated condition, comorbidities and related risk factors of the participants. Clinical characteristics also included information on the assigned treatment, such as dose, dosage, and duration; however, they were not considered in the present study, as explained in the next section. Methodological characteristics included the study location and centres, the study design and duration, the sample size, and several risk-of-bias domains concerning the internal validity of each study. These study-level aggregate clinical and methodological characteristics comprise possible effect modifiers necessary to investigate the comparability of the treatment comparisons observed in the network to conclude the plausibility of transitivity [31].

***Extraction challenges***

We endeavoured to extract as many available characteristics as possible, prioritising quantitative and qualitative characteristics. Characteristics reported in a textual format were coded where deemed feasible, namely, when there was minimal textual information similar from study to study, allowing for plausible coding of the characteristic to distinct categories.

When a study was missing all extractable characteristics, we excluded this study from the corresponding dataset. Network connectivity was not compromised in any of these networks based on the *netconnection* function of the *netmeta* R package [32]. We also excluded characteristics with missing values in all studies of the corresponding network.

Where the summarisation of quantitative characteristics was inconsistent across the studies, we used the formulas developed and evaluated by Wan and colleagues [33] to convert the results into a common descriptive measure across the studies. For instance, when most studies reported the mean age and standard deviation, and few studies reported the median age and range, we applied the following formulas to convert the median and range into mean and standard deviation, respectively:

| $\bar{x}=\frac{a+2m+b}{4}$ | formula (3) in [33] |
| --- | --- |

to obtain the mean as a function of the minimum ($a$), maximum ($b$) and median ($m$), and

| $s=\frac{b-a}{2\cdot\Phi^{-1}\left( \frac{n-0.375}{n+0.25} \right)}$ | formula (9) in [33] |
| --- | --- |

to obtain the standard deviation as a function of the minimum ($a$), maximum ($b$), the study sample size ($n$) and the inverse cumulative distribution function of the standard normal distribution.

Some systematic reviews reported aggregate participant characteristics for each treatment arm rather than the whole study. For instance, the table of characteristics of many systematic reviews reported the mean and standard deviation of participant age for each treatment arm in every study. Since effect modification refers to the interaction between treatment effect and study-level characteristics, aggregate participants' demographic and clinical characteristics reported per treatment arm must be converted to a single sensible value. For instance, we considered the weighted average of the mean age or percentage of females reported across treatment arms, with the corresponding sample sizes being the weights. If the standard deviation is also reported per treatment arm, we calculate the square root of the weighted average of the variances across the treatment arms. When the minimum and maximum of a characteristic are reported per treatment arm, we may use the formula (9) in [33] to obtain the standard deviation per treatment arm and proceed as above. At least one study-level aggregate participant-related characteristic underwent the aforementioned transformation in 115 (out of 217; 53%) datasets of our database.

Such transformations are necessary to 'mimic' a standard moderator analysis, where participant-related characteristics are aggregated at the study level. In contrast, treatment-related characteristics concerning the dose description of the compared arms, such as treatment dose, frequency, duration, interval, administration, and route, are modelled per arm, requiring specialised analysis that differs from the standard moderator analysis, including dose-response and hierarchical models [10,34]. These characteristics were not considered in our study. An exception was aggregate treatment-related characteristics, such as oral corticosteroids and prior disease-modifying anti-rheumatic drugs, measured in different amounts to the participants and reported as a percentage or mean per treatment arm. These characteristics underwent the transformations mentioned above.

**Methods B: Handing missing cases in our approach**

There is currently no guidance on addressing missing cases stemming from studies that do not report one or more of the characteristics of interest (that act as effect modifiers) when exploring the transitivity assumption. We refrained from deleting studies with at least one missing (i.e., unreported) characteristic for being counterproductive to the aims of transitivity evaluation, namely, to understand whether it is feasible to synthesise the entire evidence base using network meta-analysis. An exception was the extreme case where a study did not report any of the extracted characteristics. We also refrained from imputing missing cases using simplified approaches, such as mean imputation, as they have been highly criticised for distorting the distribution, questioning the imputed dataset's credibility. More flexible and statistically proper approaches to handling missing data deserve immediate investigation in the context of transitivity evaluation. For instance, the principal component analysis is a potential candidate, as it aligns with the unsupervised nature of our approach [35].

Nevertheless, we opted for a straightforward approach that maintains the investigated evidence base: we excluded only those characteristics missing in (i) all studies or (ii) all but one study for at least one comparison informed by at least two studies. This ensures that the within-comparison and across-comparison dissimilarities are informed by the same characteristics, allowing for comparisons across the treatment comparisons that are as 'fair' as possible.

**Methods C: Illustrate tracenma and rnmamod R packages with one example**

***Start here: tracenma R package***

We illustrate using the tracenma R package to retrieve a network with extracted study-level aggregate clinical and methodological characteristics. The official repository of tracenma (<https://github.com/LoukiaSpin/tracenma>) provides more details and examples to navigate to the functionalities and output of this R package.

Copy-paste to an R script and run the following code to load the tracenma R package and retrieve a network:

library(tracenma)

(example1 <- get.dataset(pmid = 16951908))

And this is the output: a dataset of 12 studies with seven aggregate characteristics: sex, sample.size, h.rPTH, calcium, vitamin.D, duration, and quality.

> head(example1)

$Dataset

trial treat1 treat2 arm1 arm2 sex sample.size h.rPTH calcium vitamin.D duration quality

1 Finkelstein 1998 (26) 1 2 control PTH 40 female 43 1-34 NA NA 12 2

2 Lane 1998 (42) 1 3 control PTH 25 female 51 1-34 1500 800 12 3

3 Kurland 2000 (33) 1 4 control PTH 32 male 23 1-34 1500 400 18 3

4 Cosman 2001 (24) 1 3 control PTH 25 female 52 1-34 1500 800 36 3

5 Neer 2001 (5) 1 2 control PTH 40 female 882 1-34 1000 400 21 3

6 Neer 2001 (5) 1 5 control PTH 20 female 892 1-34 1000 400 21 3

7 Neer 2001 (5) 2 5 PTH 40 PTH 20 female 878 1-34 1000 400 21 3

8 Body 2002 (43) 1 2 control PTH 40 female 146 1-34 1000 400 12 4

9 Finkelstein 2003 (28) 1 2 control PTH 40 male 48 1-34 1000 400 30 3

10 Finkelstein 2003 (28) 1 6 control PTH+ALN male 53 1-34 1000 400 30 3

11 Finkelstein 2003 (28) 2 6 PTH 40 PTH+ALN male 45 1-34 1000 400 30 3

12 Hodsman 2003 (32) 1 7 control PTH 100 female 104 1-84 500 400 12 4

13 Hodsman 2003 (32) 1 8 control PTH 75 female 105 1-84 500 400 12 4

14 Hodsman 2003 (32) 1 9 control PTH 50 female 103 1-84 500 400 12 4

15 Hodsman 2003 (32) 7 8 PTH 100 PTH 75 female 103 1-84 500 400 12 4

16 Hodsman 2003 (32) 7 9 PTH 100 PTH 50 female 101 1-84 500 400 12 4

17 Hodsman 2003 (32) 8 9 PTH 75 PTH 50 female 102 1-84 500 400 12 4

18 Orwoll 2003 (6) 1 2 control PTH 40 male 286 1-34 1000 400 11 4

19 Orwoll 2003 (6) 1 5 control PTH 20 male 298 1-34 1000 400 11 4

20 Orwoll 2003 (6) 2 5 PTH 40 PTH 20 male 290 1-34 1000 400 11 4

21 Black 2003 (23) 1 6 control PTH+ALN female 119 1-84 500 400 12 4

22 Black 2003 (23) 1 7 control PTH 100 female 179 1-84 500 400 12 4

23 Black 2003 (23) 6 7 PTH+ALN PTH 100 female 178 1-84 500 400 12 4

24 Cosman 2005 (25) 1 3 control PTH 25 female 126 1-34 1200 600 15 3

25 McClung 2005 1 5 control PTH 20 female 203 1-34 1000 400 12 3

The first column refers to the names of the included studies. The second and third columns refer to the baseline and non-baseline treatments as identifying numbers (note that the numbers in treat1 are consistently smaller than those in treat2). The fourth and fifth columns present the names of the corresponding treatments. The remaining seven columns refer to the extracted aggregate characteristics. There are more than 12 rows because there are five multi-arm studies. Therefore, the aggregate characteristics are reported for each possible pairwise comparison of each study. When a study misses a characteristic, NA appears in the corresponding part of the dataset; for instance, calcium and vitamid.D for the study Finkelstein 1998 (26).

By the way, the following code returns information about the systematic review considered to extract the data above, such as PMID number, first author, and publication year (among others):

get.dataset.index(pmid = 16951908)

***Enter the rnmamod R package***

Before we present the function to apply the approach of study dissimilarities, the dataset above needs to undergo some necessary cleaning:

# STEP 1: Turn into data.frame

dataset_new0 <- as.data.frame(example1)

# STEP 2: Remove the columns with treatment names

dataset_new <- dataset_new0[, -c(4,5)]

# STEP 3: Turn the treatment ID columns from 'double' to 'character'

dataset_new[, 2:3] <- lapply(dataset_new[, 2:3], as.character)

# STEP 4: Turn the ‘character’ characteristics into ‘integer’

dataset_new[, c(4,6)] <- lapply(dataset_new[, c(4,6)], as.factor)

Now, we move to the rnmamod R package, particularly to the comp_clustering function that applies the approach of study dissimilarities. The following code uses mostly the default arguments of the comp_clustering function and the threshold for general health setting:

library(rnmamod)

comp_clustering(input = dataset_new,

threshold = 0.13,

get_plots = TRUE)

The function returns a rich output, including results on the console and several plots. Below, we dive into the elements of the output.

Initially, a message printed in red appears on the console and pertains to the number of observed comparisons, the number of comparisons informed by one study only and characteristics that were dropped due to many missing data, rendering these characteristics unsuitable (no characteristics were dropped):

- 14 observed comparisons (8 single-study comparisons)

- Dropped characteristics: none

Then, two symmetric tables appear on the console and pertain to the study dissimilarities ($Trials_diss_table) and the overall dissimilarities for each comparison and pair of comparisons ($Comparisons_diss_table). The first table is illustrated in two separate figures:

- as a violin plot with integrated box plots and dots for the *main diagonal elements*, with each element (i.e., observed comparison) being a violin ($Within_comparison_dissimilarity), as shown below:


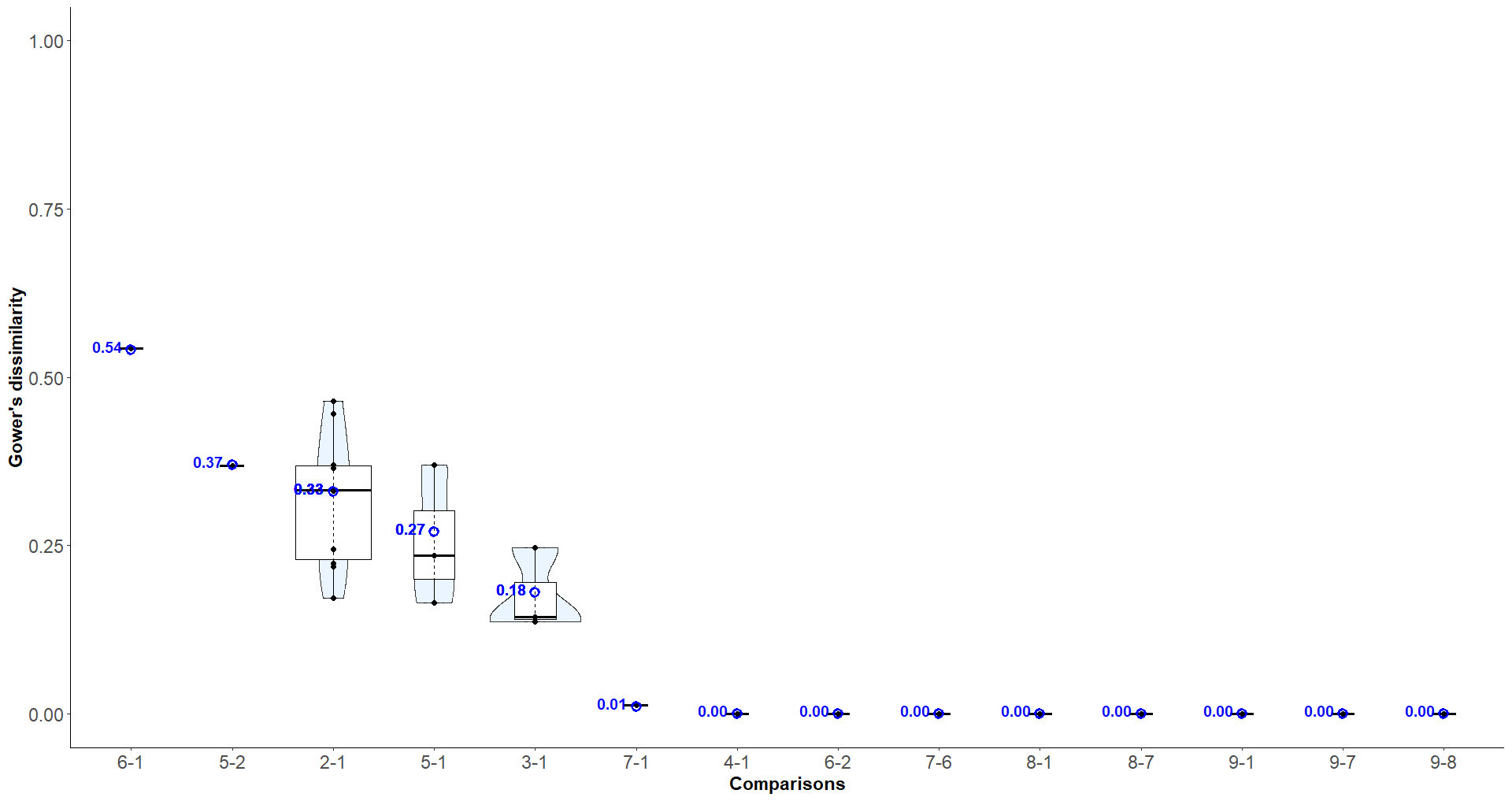


- as a violin plot with integrated box plots and dots for the *off-diagonal elements*, with each element (i.e., pair of comparisons) being a violin ($Between_comparison_dissimilarity). The plot is cluttered in this example for having too many pairs of comparisons.

The second table is illustrated as a heatmap using the selected threshold of low dissimilarity ($Dissimilarity_heatmap). The names of the observed comparisons appear on the rows and columns of the heatmap. We did not define the names of the treatments in the comp_clustering function; hence, the function used their ID number:


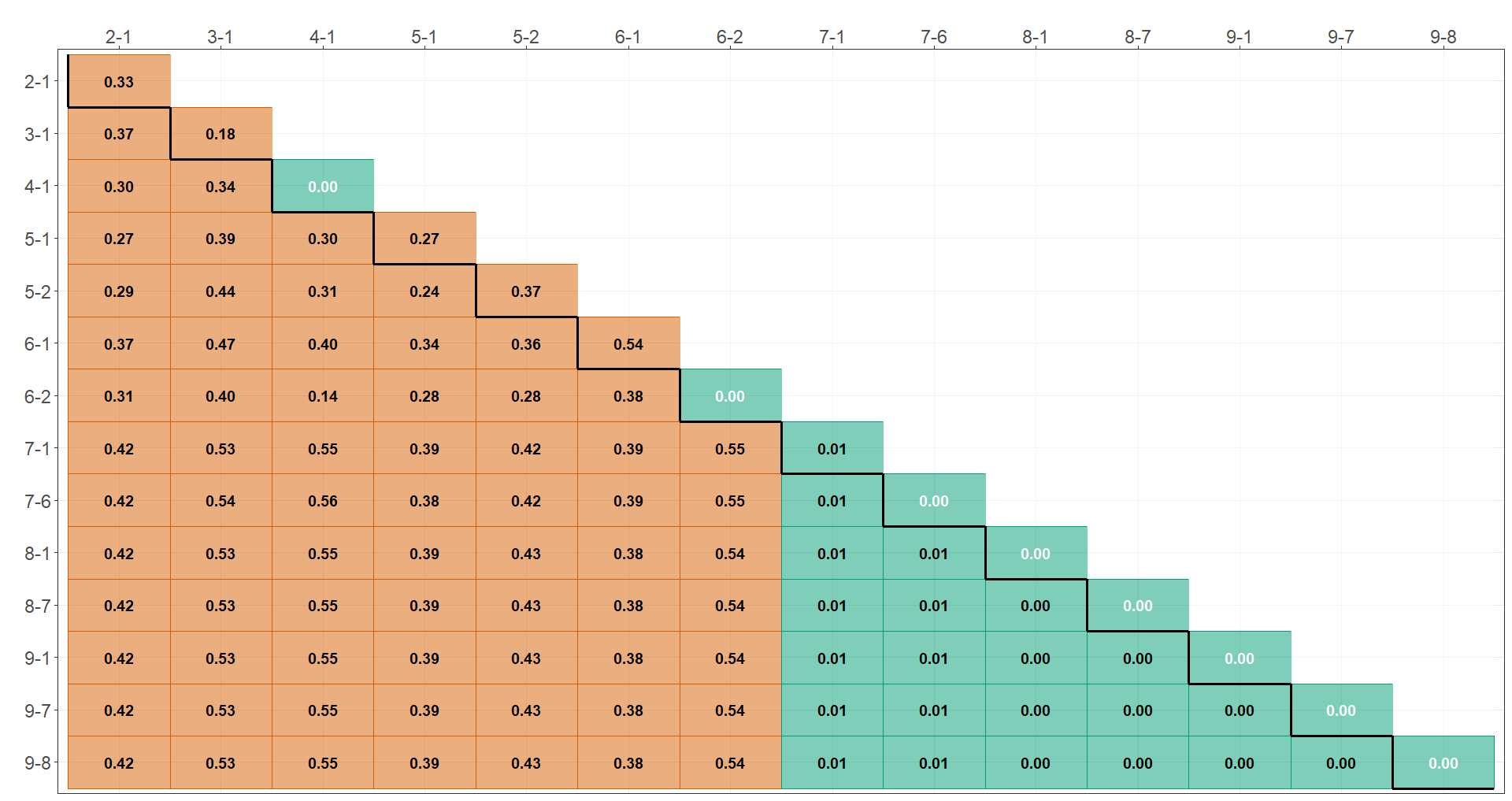


Further information in the console includes the type of the analysed characteristics ($Types_used) and the percentage of total missing data in the dataset ($Total_missing):

$Types_used

characteristic type

1 Dataset.sex integer

2 Dataset.sample.size double

3 Dataset.h.rPTH integer

4 Dataset.calcium double

5 Dataset.vitamin.D double

6 Dataset.duration double

7 Dataset.quality double

$Total_missing

[1] "1.14%"

- Visit the documentation of the comp_clustering function to learn more about the arguments of the functions: <https://loukiaspin.github.io/rnmamod/reference/comp_clustering.html>.
