# Supplementary material for: An empirical study on 209 networks of treatments revealed intransitivity to be common and multiple statistical tests suboptimal to assess transitivity
Source: BMC Med Res Methodol. 2024 Dec 16;24:301. doi: 10.1186/s12874-024-02436-7 (PMC11648297; doi:10.1186/s12874-024-02436-7)
Supplement: Supplementary file 3 — Additional file 3: Figure S1. Grouped bar plots on the distribution of the different outcome types and treatment-comparator types in 214 datasets referring to networks of treatments for the primary outcomes. Figure S2. Dot plots with integrated interval bars on the number of extracted characteristics from 214 datasets (plot a)) and the reduced set of 209 datasets (plot b)). Figure S3. Violin plots with integrated interval bars on the Gower's dissimilarity coefficient for each combination of outcome type with treatment-comparator type. Figure S4. Violin plots with integrated interval bars on the within-comparison dissimilarities for each combination of outcome type with treatment-comparator type. Figure S5. Grouped bar plots on the percentage of treatment comparisons with 'low' and 'likely concerning' within-comparison dissimilarity based on two thresholds of low dissimilarity. Figure S6. Violin plots with integrated interval bars on the between-comparison dissimilarities for each combination of outcome type with treatment-comparator type. Figure S7. Grouped bar plots on the percentage of pairs of treatment comparisons with 'low' and 'likely concerning' between-comparison dissimilarity based on two thresholds of low dissimilarity. [file 12874_2024_2436_MOESM3_ESM.docx]

**Additional file 3**

**Supplementary material for the manuscript entitled 'An empirical study on 209 networks of treatments revealed intransitivity to be common and multiple statistical tests suboptimal to assess transitivity'**

Loukia M. Spineli^1^

^1^Midwifery Research and Education Unit, Hannover Medical School, Hannover, Germany


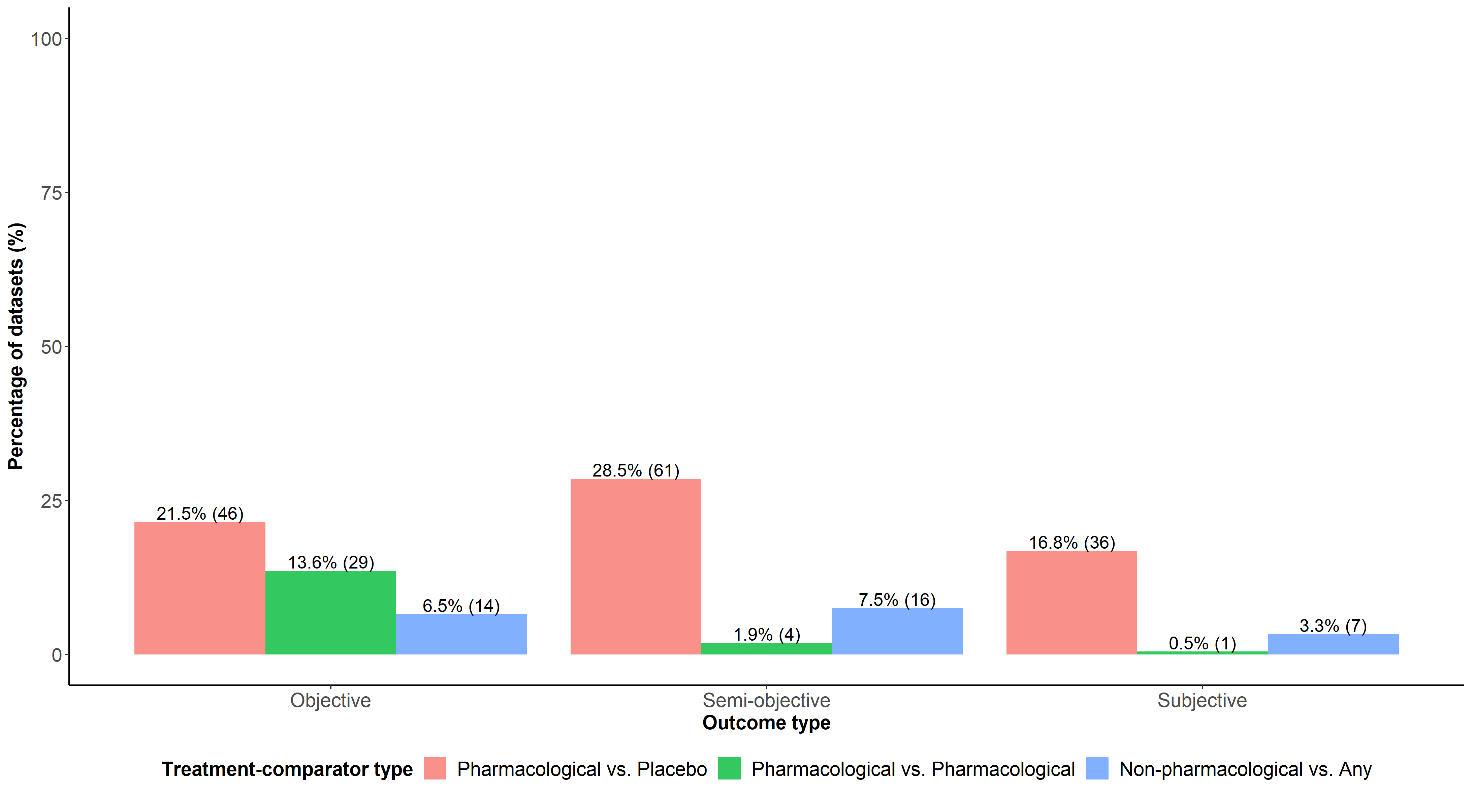


**Figure S1.** Grouped bar plots on the distribution of the different outcome types and treatment-comparator types in 214 datasets referring to networks of treatments for the primary outcomes. Percentages are calculated out of the total 214 datasets.


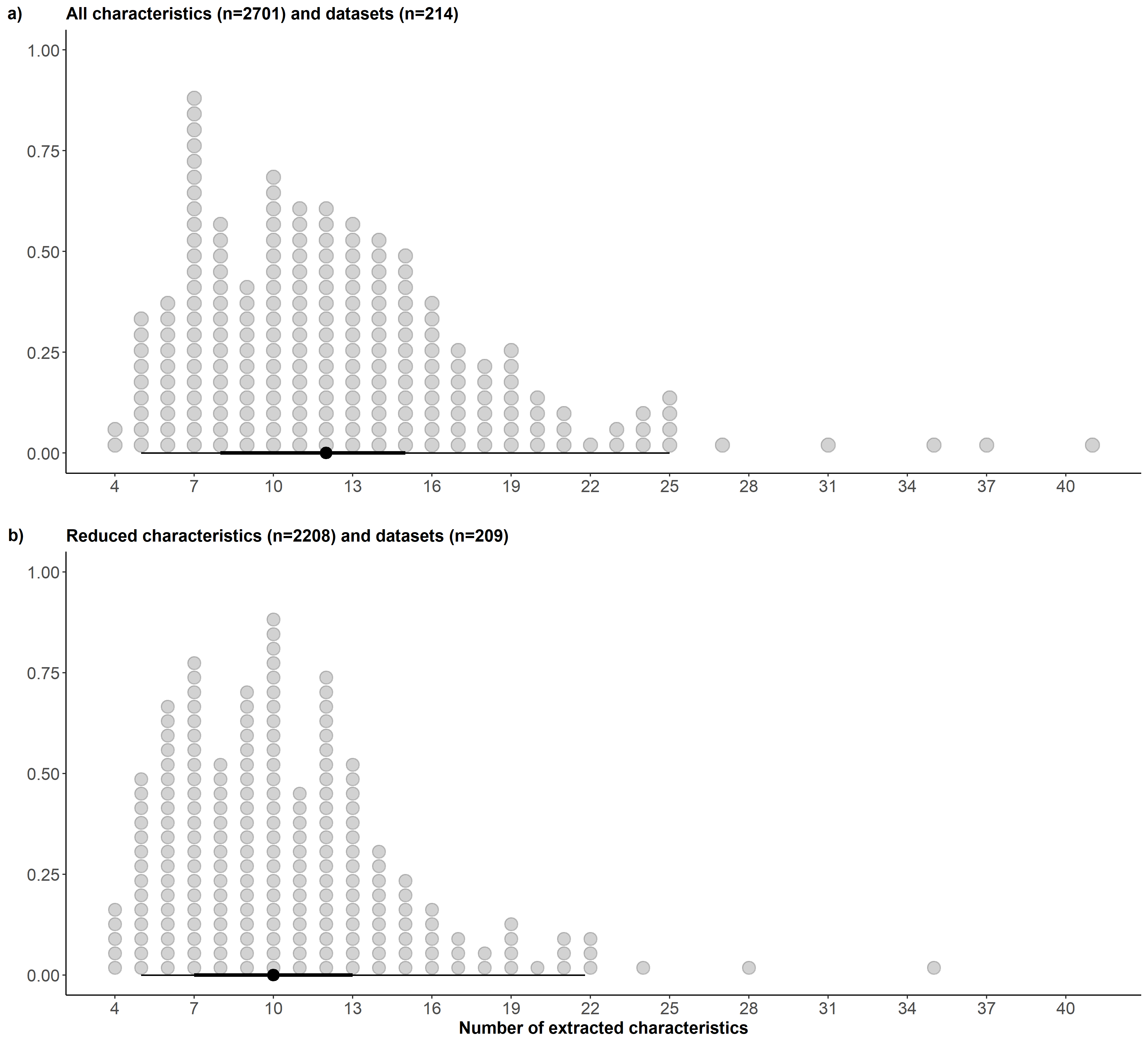


**Figure S2.** Dot plots with integrated interval bars on the number of extracted characteristics from 214 datasets (plot a)) and the reduced set of 209 datasets (plot b)). The overlapping, horizontal, black interval bars below the dots refer to the interquartile range (thick black line) and 95% interval (thinner black line). The black point on the interval bars refers to the median. After dropping characteristics with too many missing data, five datasets were removed for containing less than four characteristics, leading to 209 datasets for analysis (plot b)).


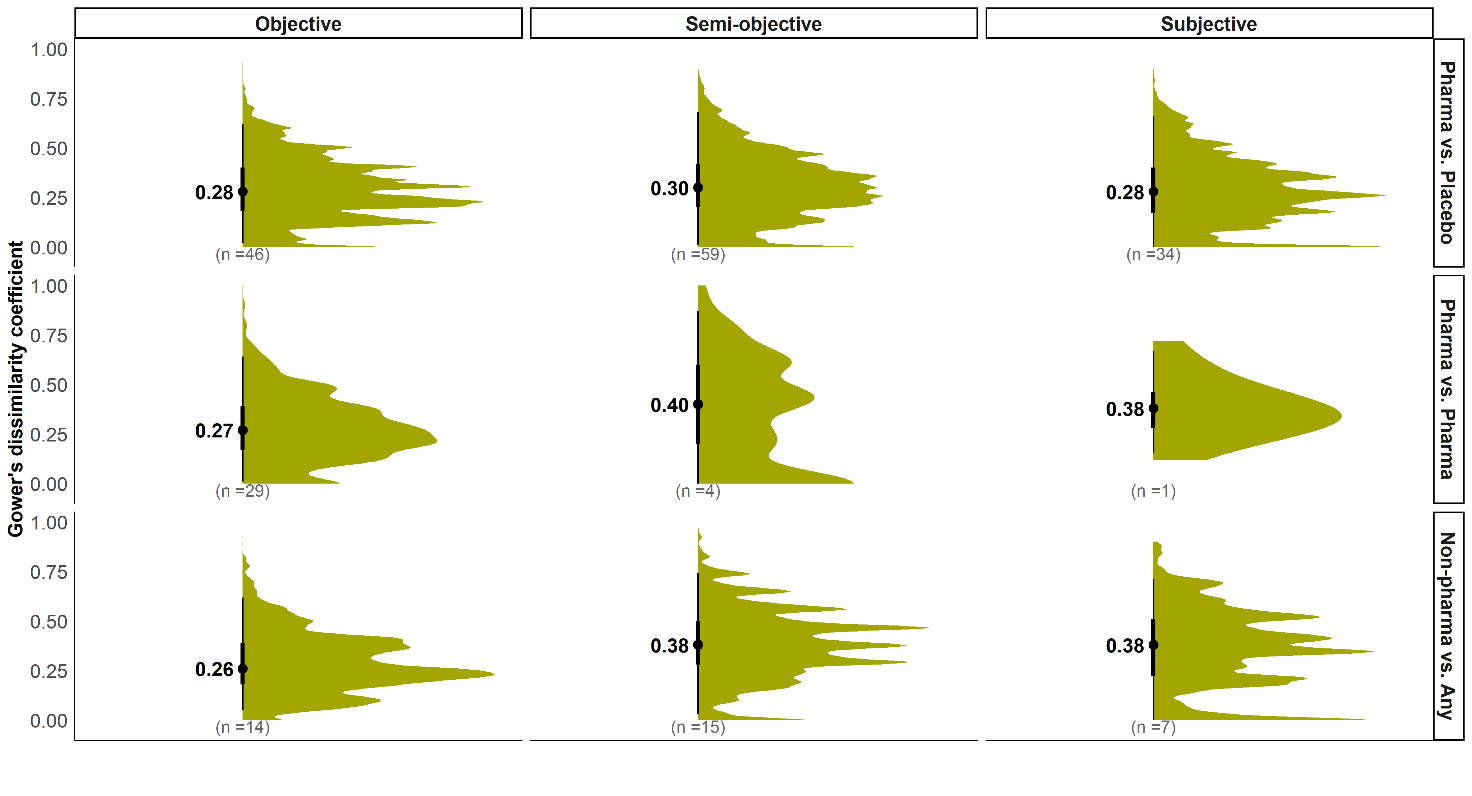


**Figure S3.** Violin plots with integrated interval bars on the Gower's dissimilarity (GD) coefficient for each combination of outcome type (objective, semi-objective, and subjective) with treatment-comparator type (pharmacological versus placebo/control, pharmacological versus pharmacological, and non-pharmacological versus any). A total of 209 datasets were considered. The black numbers refer to the median GD value, the thick vertical interval bars to the interquartile range and the thin vertical interval bars to the 95% interval. The grey numbers in parenthesis at the bottom refer to the number of datasets that belong to the corresponding combination. Pharma, pharmacological


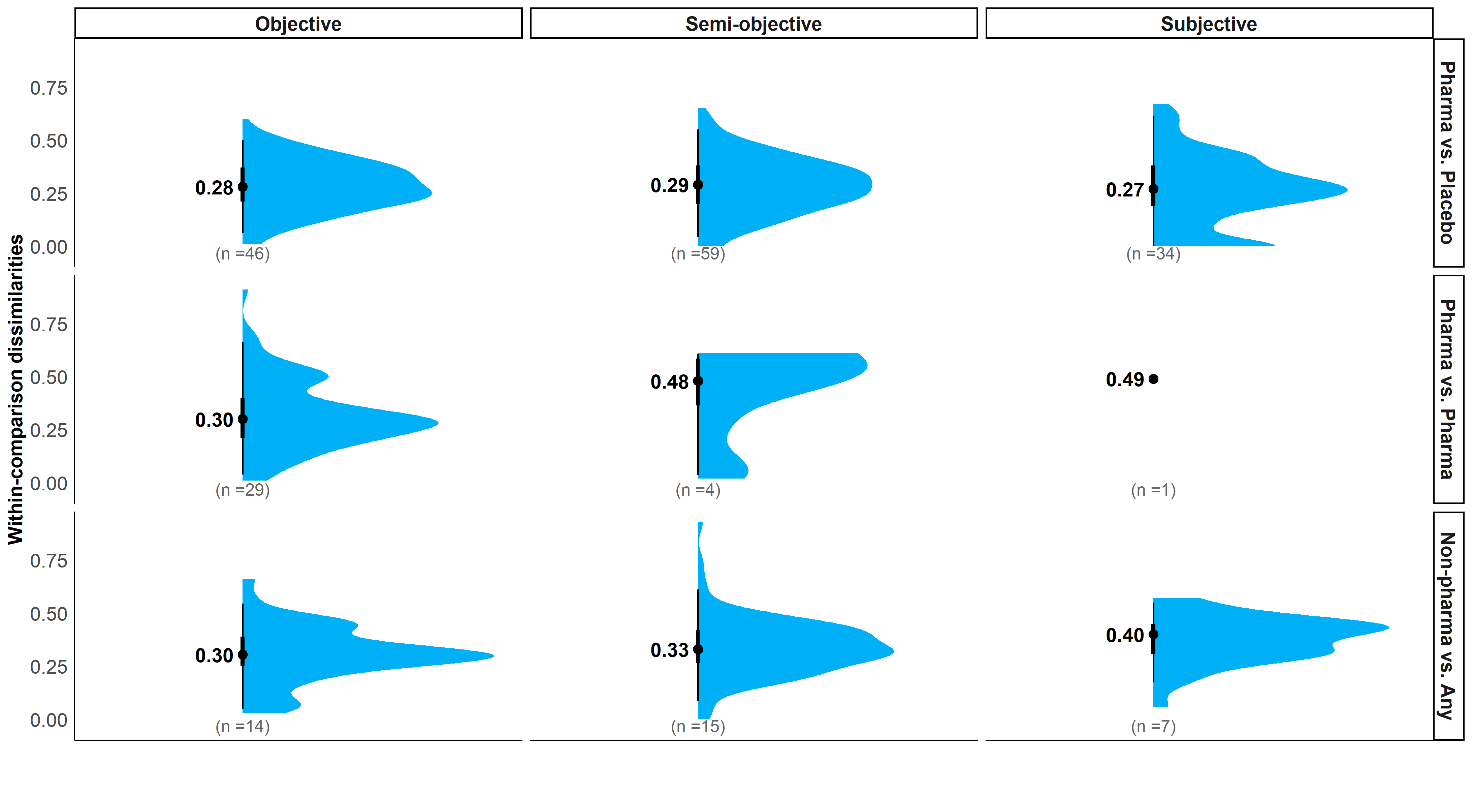


**Figure S4.** Violin plots with integrated interval bars on the within-comparison dissimilarities for each combination of outcome type (objective, semi-objective, and subjective) with treatment-comparator type (pharmacological versus placebo/control, pharmacological versus pharmacological, and non-pharmacological versus any). A total of 209 datasets were considered. The single dataset with a subjective outcome and pharmacological treatments contained only one non-single-study comparison, yielding one value of within-comparison dissimilarity. The black numbers refer to the median within-comparison dissimilarity, the thick vertical interval bars to the interquartile range and the thin vertical interval bars to the 95% interval. The grey numbers in parenthesis at the bottom refer to the number of datasets that belong to the corresponding combination. Pharma, pharmacological


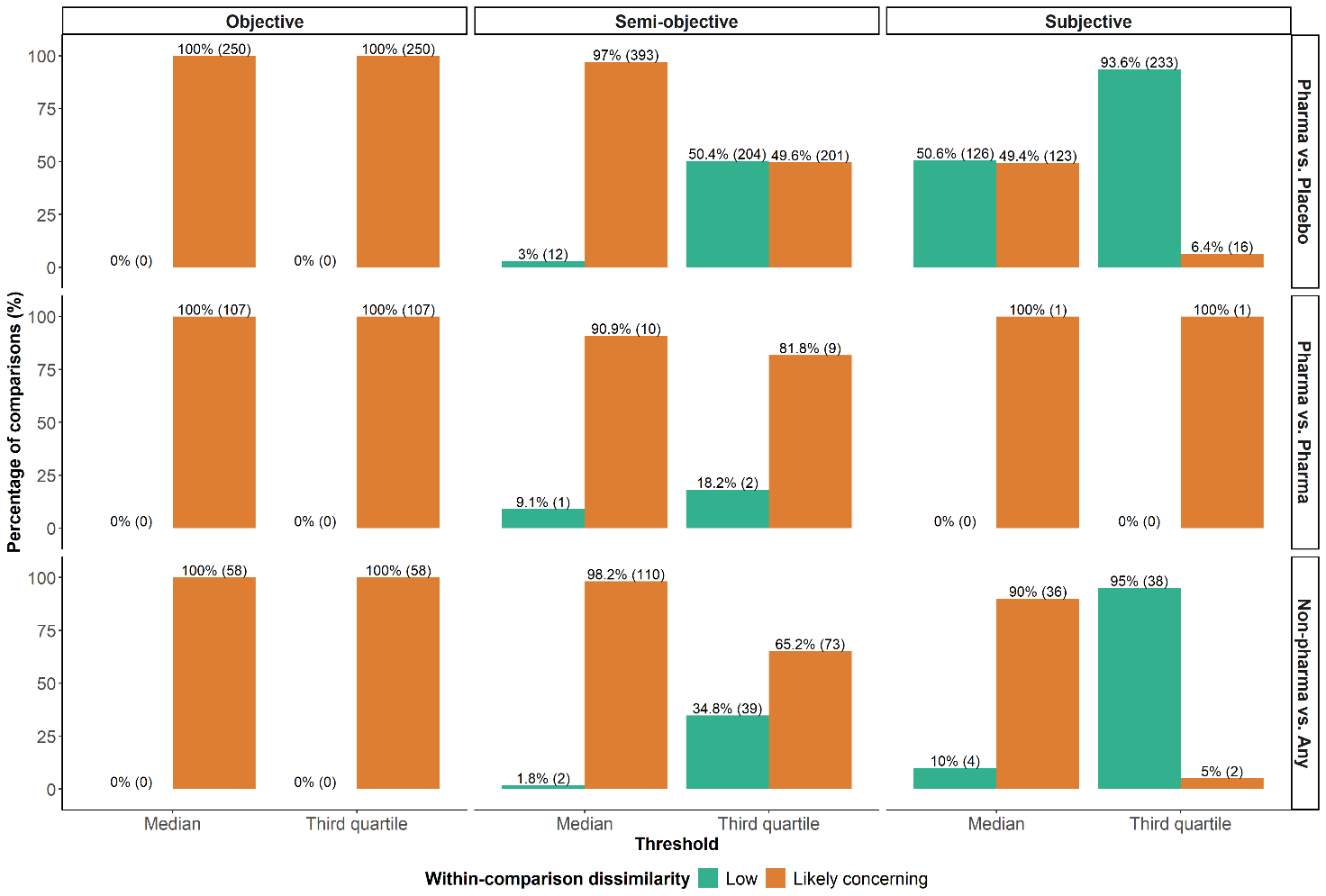


**Figure S5.** Grouped bar plots on the percentage of treatment comparisons with 'low' (green bars) and 'likely concerning' (orange bars) within-comparison dissimilarity based on two thresholds of low dissimilarity: the median and third quartile of the selected predictive distribution for $I^{2}$. The thresholds are tailored to the outcome type (objective, semi-objective, and subjective), treatment-comparator type (pharmacological versus placebo/control, pharmacological versus pharmacological, and non-pharmacological versus any) and average sample size of the trials informing the datasets (small, moderate, and large). A total of 209 datasets were considered. The numbers in parentheses refer to the number of corresponding treatment comparisons.


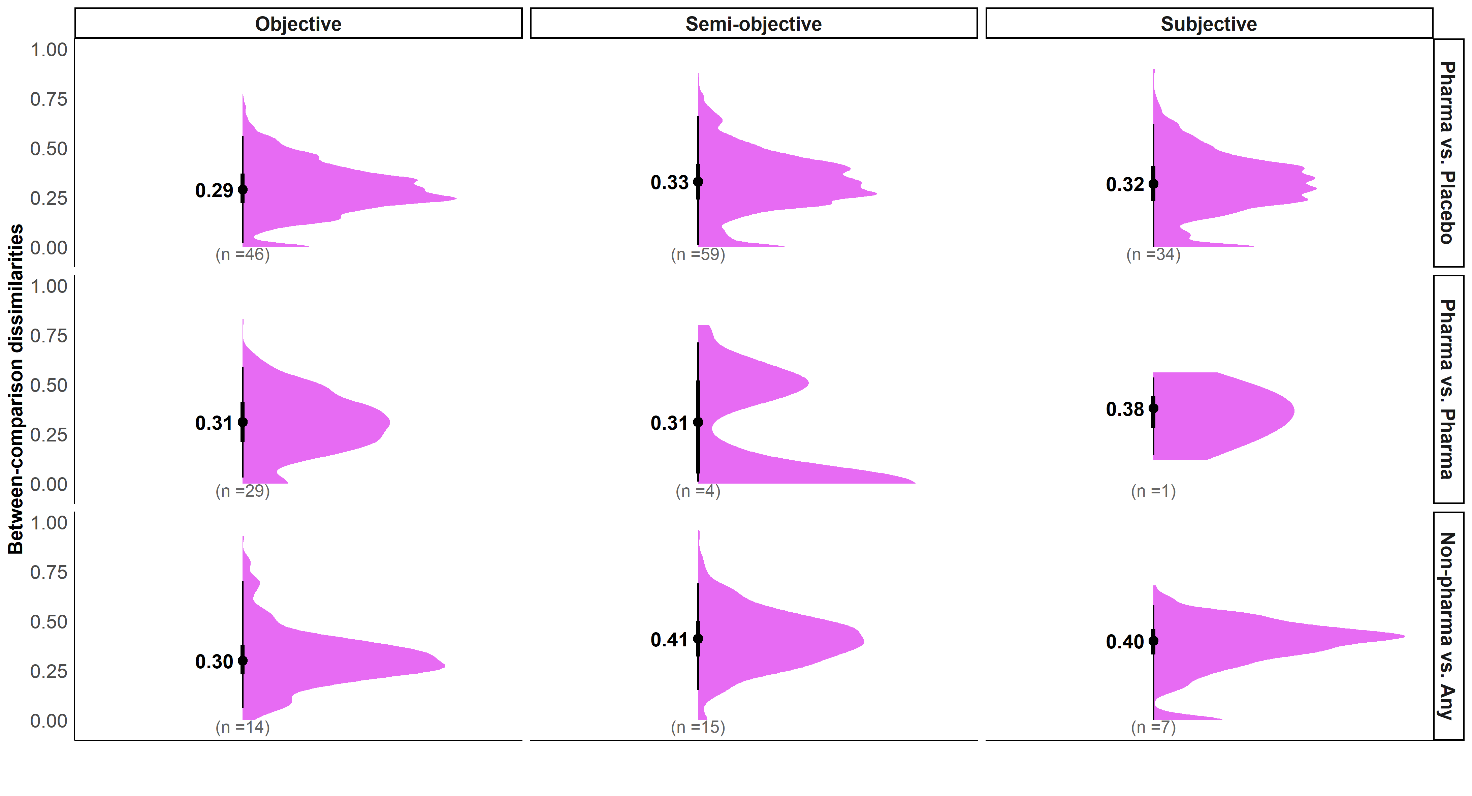


**Figure S6.** Violin plots with integrated interval bars on the between-comparison dissimilarities for each combination of outcome type (objective, semi-objective, and subjective) with treatment-comparator type (pharmacological versus placebo/control, pharmacological versus pharmacological, and non-pharmacological versus any). A total of 209 datasets were considered. The black numbers refer to the median between-comparison dissimilarity, the thick vertical interval bars to the interquartile range and the thin vertical interval bars to the 95% interval. The grey numbers in parentheses at the bottom refer to the number of datasets that belong to the corresponding combination. Pharma, pharmacological


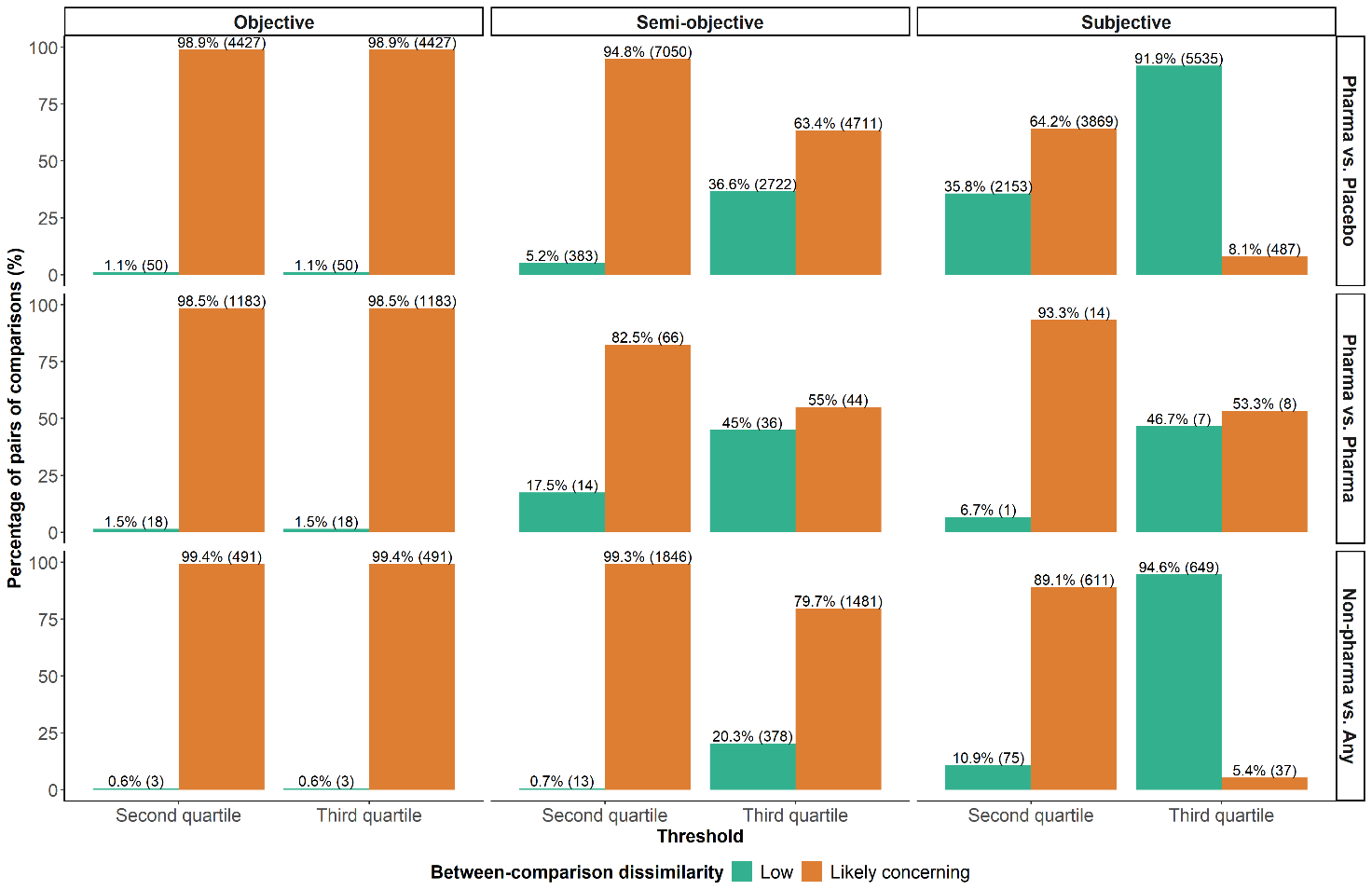


**Figure S7.** Grouped bar plots on the percentage of pairs of treatment comparisons with 'low' (green bars) and 'likely concerning' between-comparison dissimilarity (orange bars) based on two thresholds of low dissimilarity: the median and third quartile of the selected predictive distribution for $I^{2}$. The thresholds are tailored to the outcome type (objective, semi-objective, and subjective), treatment-comparator type (pharmacological versus placebo/control, pharmacological versus pharmacological, and non-pharmacological versus any) and average sample size of the trials informing the datasets (small, moderate, and large). A total of 209 datasets were considered. The numbers in parentheses refer to the corresponding pairs of treatment comparisons. Pharma, pharmacological
